# Digital channel–enabled distributed force decoding via small datasets for hand-centric interactions

**DOI:** 10.1126/sciadv.adt2641

**Published:** 2025-01-22

**Authors:** Yifeng Tang, Gen Li, Tieshan Zhang, Hao Ren, Xiong Yang, Liu Yang, Dong Guo, Yajing Shen

**Affiliations:** ^1^ The Robot and Automation Center and the Department of Biomedical Engineering, City University of Hong Kong, Hong Kong, 999077, China.; ^2^ Department of Electronic and Computer Engineering, Hong Kong University of Science and Technology, Hong Kong, 999077, China.; ^3^ Center for Smart Manufacturing, Hong Kong University of Science and Technology, Hong Kong, 999077, China.

## Abstract

Tactile interfaces are essential for enhancing human-machine interactions, yet achieving large-scale, precise distributed force sensing remains challenging due to signal coupling and inefficient data processing. Inspired by the spiral structure of *Aloe polyphylla* and the processing principles of neuronal systems, this study presents a digital channel–enabled distributed force decoding strategy, resulting in a phygital tactile sensing system named PhyTac. This innovative system effectively prevents marker overlap and accurately identifies multipoint stimuli up to 368 regions from coupled signals. By integrating physics into model training, we reduce the dataset size to just 45 kilobytes, surpassing conventional methods that typically exceed 1 gigabyte. Results demonstrate PhyTac’s impressive fidelity of 97.7% across a sensing range of 0.5 to 25 newtons, enabling diverse applications in medical evaluation, sports training, virtual reality, and robotics. This research not only enhances our understanding of hand-centric actions but also highlights the convergence of physical and digital realms, paving the way for advancements in AI-based sensor technologies.

## Teaser

Introducing digital channels enables distributed hand force mapping just using ultrasmall datasets.

## INTRODUCTION

The tactile interface is one of the most critical elements in intelligent human-machine interactions, allowing us to acquire large-scale tactile information from the surroundings and further give feedback to the machines (*1*–*5*). As one of the most direct physical extensions of our consciousness, our hands serve as the primary source of nuanced tactile sensations and function as intelligent tools for crafting and using objects in the physical realm (*6*). In addition, hand-centric interaction devices, such as joysticks, mice, keyboards, and touchpads, are also regarded as a major method to bridge the gap between human beings and the virtual world (*7*, *8*). Regrettably, despite the critical role of the hand, decoding the strength and distribution of the forces generated by hands is still challenging, which has significantly impeded progress in various fields, such as precise medical treatment (*9*), efficient sports training (*10*), virtual reality (VR) manipulation (*11*, *12*), robotics (*13*–*15*), and more.

For centuries, the hydraulic, pneumatic, and mechanical dynamometers (*16*) were the commonly used methods for assessing the force exerted by the human hand, but they can only provide information of maximum force, lacking spatial and temporal details (table S1). Recent advances in soft tactile skin provide opportunities for capturing force distribution, primarily through array-based [piezoresistive (*17*, *18*), capacitive (*2*, *15*, *19*), piezoelectric (*20*), triboelectric (*12*, *21*), magnetic (*14*), etc.] transductions and vision-based [Gelsight (*22*, *23*), Tactip (*24*), TacLINK (*25*, *26*), Insight (*27*), etc.] techniques. However, the electrical wire array–based method faces reliability issues due to material’s sensitivity to environmental factors (temperature, humidity, electrical and magnetic fields) (*14*, *28*, *29*) and cross-talk problems (*15*, *21*, *25*), leading to reduced accuracy in large-scale contacts. While the optical-based method offers higher robustness by eliminating messy wires, they encounter accuracy issues in multiple large-areas contacts (*25*, *27*, *30*) and have a limited sensing range, often hindered by easily occluded markers (*22*, *24*–*26*, *31*, *32*) and/or usually requiring massive datasets (*26*, *27*, *33*). Moreover, the information obtained from almost all these soft tactile sensors is inherently the analog signals coupled from several unknown loading sources, making the force decoding very complicated, especially for forces applied from multiple points over a large area, just as hand grip force.

**Fig. 1.**
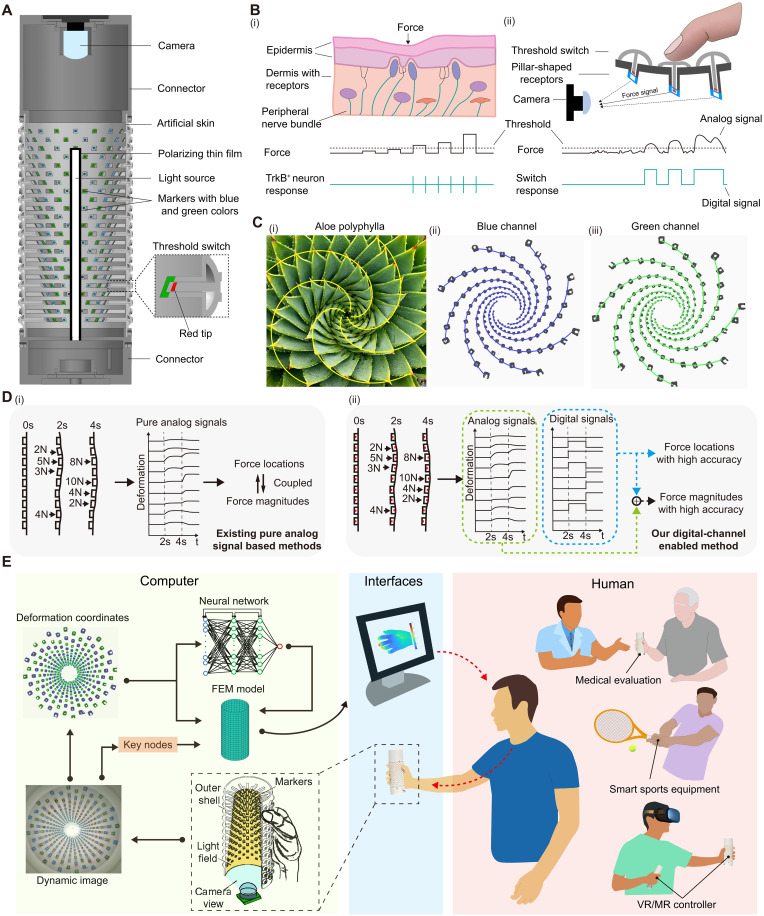
Schematic illustration of the digital channel enabled phygital tactile system for large scale and precise distributed force sensing with ultrasmall datasets. (**A**) Cutaway view of the overall structures and components of the PhyTac. [(**B**), i] The structure of normal human skin and the deformation when force presses the skin (top). The illustration of TrkB^+^ neuron response when force is applied (bottom). [(B), ii] The detailed structure of the outer shell and the deformation when the finger presses the outer shell. Only the middle threshold switch is activated (top). The illustration of switch response and the generation of digital signals when force is applied (bottom). [(**C**), i] The spiral phyllotaxis of the *Aloe polyphylla*. Photo courtesy of C. Thorogood. [(C), ii] The biomimetic spiral arrangement of markers in the blue channel. [(C), iii] The biomimetic spiral arrangement of markers in the green channel. (**D**) Comparison of conventional pure analog signal methods and our digital channel enabled method. Force locations and force magnitudes are coupled with each other in pure analog signals [(D), i]. Force locations are decoded by digital signals with high accuracy and help reconstruct force magnitudes [(D), ii]. (**E**) PhyTac for human-machine interaction by measuring the hand force distribution, such as for medical evaluation, smart sports training, and VR.

This study introduces a digital channel–enabled hand force sensing and processing strategy and then develops a phygital tactile sensing system (PhyTac) inspired by the decoding principles of touch neuronal systems (*34*, *35*) and the spiral structure found in leaves of rosette plants (*36*–*39*) (*Aloe polyphylla*, *Aloe juvenna*, etc.) (Fig. 1). We achieve the highest marker density on record in parallel-type sensing surfaces and successfully decode the location of the loading [key nodes of interest (KOI)] from coupled mass signals, offering physically meaningful high-quality data. Our proposed physical model–enhanced neural network (FEM-NN) demonstrates the capability to reconstruct the distributed force map with high accuracy (0.11 N, 97.7% accuracy) within a large sensing range (0.5 to 25 N for a single point) using just a small dataset (45 KB). The capability of PhyTac in constructing the spatial-temporal hand force maps enables versatile applications, including finger and palm force evaluation, dynamic hand monitoring in tennis playing, VR manipulation, and human-robot interaction.

## RESULTS

### Bioinspired digital channel and spiral maker arrangement

The PhyTac (Fig. 1 and fig. S5) primarily comprises a bioinspired outer shell with receptor arrays, a polarizing linear light source positioned along the central axis, a top-mounted camera for motion capture, and connectors facilitating the integration of all components. The outer shell is cylindrically shaped to facilitate interaction, as the cylinder closely approximates the form of most handles in human life (*40*). To enhance reliability and robustness, we opt for optical signals as the sensory field to decode force-caused deformation, thereby eliminating the need for cumbersome wire arrays and rendering them minimally susceptible to environmental changes, such as temperature and humidity (*41*). The light source consists of a linear chip-on-board (COB) light strip with covered polarizing film, effectively eliminating unwanted refraction and uniformly projecting light onto all markers (fig. S6). Each receptor on the outer shell is designed as an independent unit, sensing the force applied in its vicinity. The extended height of each receptor gradually increases (ranging from 0.5 to 11.5 mm) away from the camera to prevent image blocking along the depth direction (Fig. 1A).

The state-of-the-art soft tactile sensors (*12*, *14*, *18*, *25*, *27*) face challenges in acquiring accurate force information when the load is applied on multiple points in a large area (Fig. 1Di), even massive datasets and complex algorithms are used. The reason is that the load-caused deformation in the surrounding area is quite complex, always resulting in coupled analog signal mixing by several unknown loading sources (locations, magnitudes, etc.), as illustrated in Fig. 1Di. The TrkB^+^ is a kind of sensory neuron in skin, producing spiking signal that exhibits on and off responses to tactile indentation (*34*, *35*). This on-off digital property allows TrkB^+^ to lonely encode small threshold forces and help localize the stimulus. Inspired by the working principle of TrkB^+^ neurons, we deploy soft bowl-shaped threshold switches on the outer shell of PhyTac to output “on” and “off” digital signals (Fig. 1Bii), which can not only encode threshold forces but also help localize the loading points. When the multiple-point load is applied on the PhyTac, only the loading point will activate the switch to extend the red tip although numerous receptors are being deformed (Fig. 1B, i and ii). Then, the corresponding receptors to the activated switch will be counted as the KOI denoting the load location. Consequently, by taking the location of the applied force as preknowledge, the force magnitudes can be calculated more easily and accurately, as illustrated in Fig. 1Dii. The criterion of threshold is to filter out unwanted disturbances from surrounding stimuli. In this case, we set a force threshold of 0.5 N for the switch through mechanical design. Note that the threshold is adjustable for different applications.

Recognizing and tracking motion objects in three-dimensional (3D) space is another formidable challenge in image processing due to static and dynamic occlusions of light paths, as well as nonuniform light environments with highlights, shadows, etc. (*42*). For camera-based sensors, despite the perpendicular-type sensing surface (perpendicular to the optical axis of camera) can have a high marker density, the marker density of parallel-type sensing surfaces is still low due to easily occluded markers (fig. S7), resulting in small sensing range and limited resolution. For the measurement of the hand force, the sensing surface should be parallel to the optical axis, in which case it is very difficult to increase marker density. Phytologists disclosed that the spiral pattern of rosette plants, such as *Aloe polyphylla*, is selected by evolution to avoid light path occlusion and maximize sunlight capture benefits (*36*–*39*). This smart strategy can optimize the light path arrangement by avoiding leaf occlusion or self-shading, which is very instructive to the camera-based sensor design (fig. S8). To tackle the marker occlusion problem in 3D space, we lay out the receptors in a 3D spiral distribution, mimicking the leaf structure of *Aloe polyphylla* (Fig. 1C). In addition, by incorporating color distinction—using two spirals with blue and green colors, respectively—we achieve a remarkable marker density up to 1.63/cm^2^, the highest record by far in vision-based sensors with parallel-type sensing surfaces (table S2). Furthermore, even with such a high density, we successfully avoid marker fusion at different depths during motion, achieving high-accuracy tracking with a root mean square error (RMSE) of only 0.18 mm.

Figure 1E illustrates the comprehensive working process of PhyTac as the interactive interface, encompassing the reception of the hand force signal to the output signal, bridging the real and virtual world. Briefly, when the PhyTac receives the force from the hand, its outer shell undergoes deformation, and the motion of each receptor is captured by the camera. Subsequently, leveraging the identified key nodes and the displacements of all markers as input, the FEM-NN accurately establishes the mapping relationship between marker displacements and force distribution. This process allows the system to obtain the reconstructed tactile force information from the hand, ultimately facilitating advanced human-machine interaction across a broad spectrum of applications, such as evaluating the functionalities of muscles/nerves/bones of hands in clinical medicine, testing distributed force loading in sports, and manipulating objects precisely in the virtual world among other possibilities.

### Distributed force decoding principle and process

**Fig. 2.**
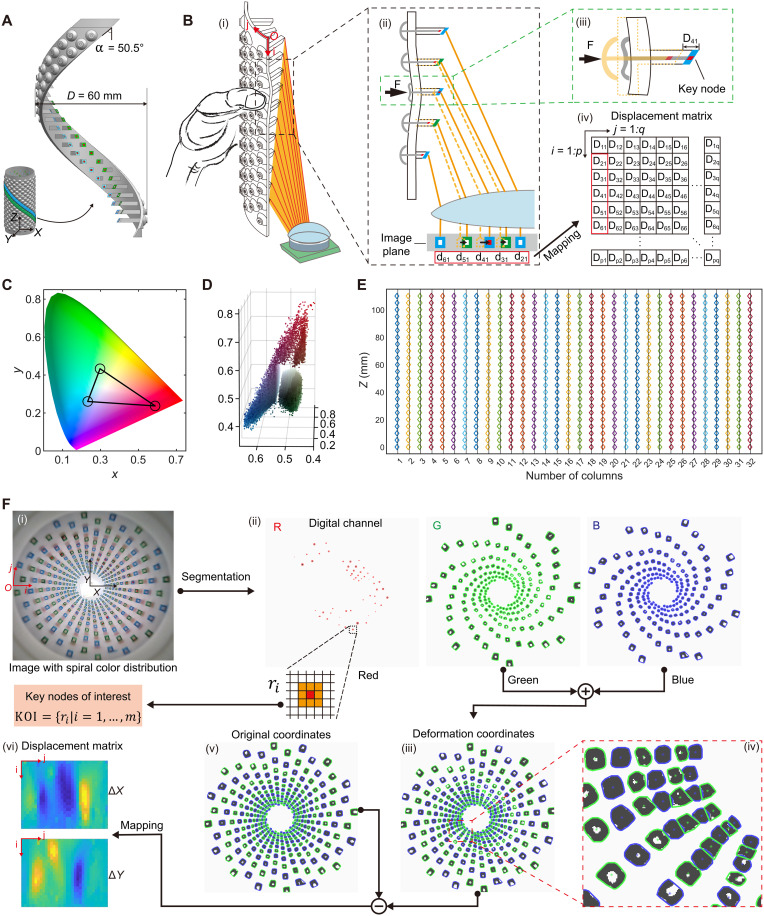
Design and working principle of the PhyTac with spirally arranged receptors and digital channels. (**A**) The minimum spiral element of the PhyTac. (**B**) Schematic illustration showing the relationship between applied force and displacement matrix of markers. [(B), i] Cutaway view of the PhyTac measuring the hand grip force. [(B), ii] Only the receptors at the loading point will be activated though several receptors are deformed. [(B), iii] Enlarged image of the activated receptors, named KOI. [(B), iv] Mapping of displacement matrix. (**C**) The selected colors in chromaticity diagram. (**D**) Point cloud in YCbCr color space. (**E**) Original coordinates unwrapped in the circumferential direction. (**F**) The procedures of data processing. [(F), i] The image with spiral color distribution. [(F), ii] Segmented red channel containing the digital information of the force location (KOI), and the blue and green channels containing the information of marker displacement. [(F), iii] Deformation coordinates of all markers. [(F), iv] Enlarged image shows the spiral arrangement can address marker fusion problem and segment overlapped markers effectively. [(F), v] Original coordinates of all markers. [(F), vi] The displacement matrix in *X* and *Y* directions.

Figure 2A presents the schematic of the two-column spirally arranged markers (spiral angle α: 50.5°, radius R: 30 mm) colored blue and green. When the hand grasps the PhyTac (Fig. 2B), the outer shell’s surface deforms, and the surrounding receptors generate displacement accordingly. To represent the marker’s motion, we establish a 2D coordinate system at point O, with i as the axial direction and j as the circumferential direction (Fig. 2Bi). The markers’ displacements in the image plane presented, denoted as $d_{\mathrm{ij}}$ (Fig. 2Bii), can be measured through image processing and then translated into real displacement $D_{\mathrm{ij}}$ (Fig. 2Biii) in the world coordinate system *XYZ* (Fig. 2A). Consequently, the complete displacement matrix D can be obtained as illustrated in Fig. 2Biv. The chromaticity diagram (CIE 1976) and point cloud in the YCbCr color space demonstrate that the selected three distinctly different colors (blue, green, and red) are well-separated in the digital color space, facilitating easy segmentation in image processing (Fig. 2, C and D).

When the local force surpasses the threshold, the red tips of the threshold switch extend outward and are identified through the red channel (Fig. 2F and movie S1). The markers, whose threshold switches are activated in the red channel, are signified as logic “1,” and the other markers are signified as logic “0,” forming a digital channel that represents the spatial distribution of critical force. This digital channel has a binary form: $b_{1}b_{2}\ldots .b_{n}$, where b is a binary number 0 or 1, and n represents the total number of nodes. Then, all logic “1” nodes are defined as KOI: $=\left\{r_{i}|i=1,\ldots ,m\right\}$, where $r_{i}$ is the index of key node and m is the number of key nodes. During the deformation process, although spatial distribution of force changes every frame, the digital channel can obtain the spatial distribution from coupled analog signals efficiently, assuring the accuracy of force estimation and reducing computational resources when there is no logic “1” detected. By contrast, the pure analog signal–based methods need complex algorithms and massive datasets to decode force locations, not only facing challenges in large-scale contacts but also consuming more computational resources.

Simultaneously, the positions of all markers in the image coordinate are recognized through the green and blue channels (Fig. 2Fiii) and are dynamically tracked by using a k-medoids–based coordinate matching method (fig. S9). Subsequently, the marker coordinates on the 3D outer shell are constructed using a coordinate geometrical mapping function (fig. S10 and note S1). Consequently, the displacement matrix D is obtained by accounting for the difference between initial coordinates and deformed coordinates (Fig. 2Fvi), providing the magnitude distribution of all forces.

It is also noteworthy that the bioinspired spiral structure allows the camera to capture to the maximum extent by preventing marker fusion. This feature is beneficial as it ensures clear segmentation of markers and accurate recognition of corresponding coordinates. As illustrated in Fig. 2Fiv, even when marker overlap occurs due to high marker density and large deformation, the bioinspired spiral structure enables clear segmentation of marker contours. For comparison, markers fuse together when using a traditional structure (fig. S11), leading to coordinate errors and force inaccuracies. Experimental results demonstrate that the precision of reconstructing original coordinates in the initial image is up to 0.18 mm (RMSE), as shown in Fig. 2E. This indicates that the reconstructed coordinates (diamond marks) almost coincide with the reference coordinates (black line).

### Physical model–enhanced neural network

Machine learning has proven to be a powerful method for image-based force construction (*22*, *27*, *33*), leveraging its strength in nonlinear fitting, end-to-end mapping procedures, etc. However, in addition to big data processing caused inefficiency, another significant drawback of this type of data-driven approach is the absence of underlying physics. Even with extensive data, it may yield arbitrary extrapolating when faced with situations beyond the scope of the training data, because, in real practice, it is difficult to collect comprehensive training data for complex contacts involving different contact locations and magnitudes. Therefore, we propose an approach integrating the mechanical model of the outer shell into neural network training, named FEM-NN, aiming to circumvent the need for massive datasets and mitigate inaccuracies arising from arbitrary extrapolation.

**Fig. 3.**
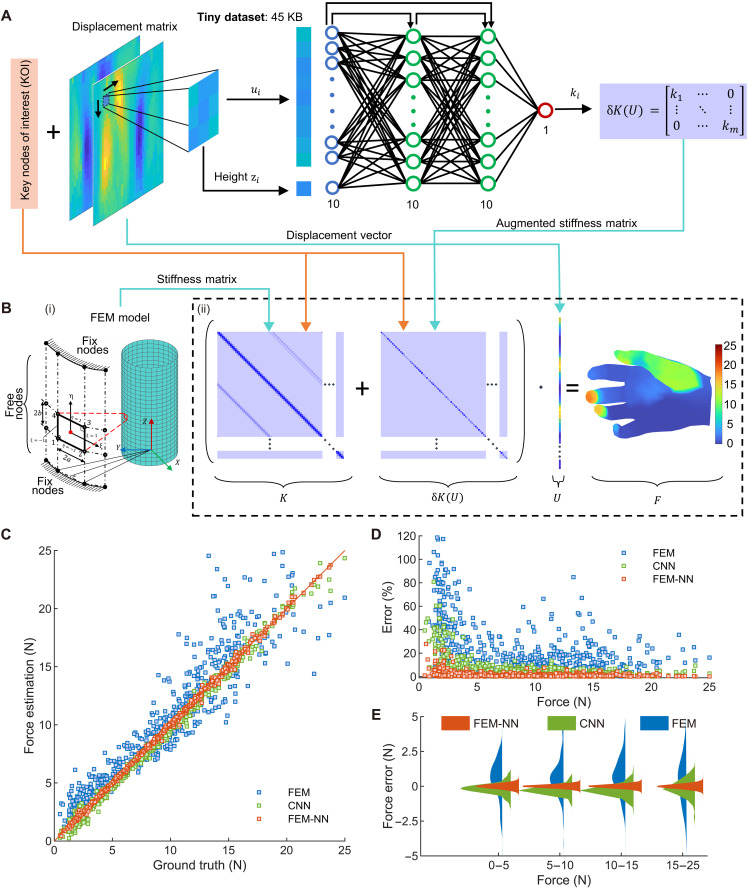
Principle of FEM-NN and its superiorities in small dataset and high accuracy. (**A**) The architecture of FEM-NN with tiny dataset (45 KB). The inputs are KOI and displacement matrix, and the output is augmented stiffness matrix. (**B**) FEM-NN based force reconstruction algorithm. [(B), i] The finite element model of the PhyTac, including the geometry of four-node rectangular element (left) and the mesh topology (right). [(B), ii] The force reconstruction algorithm, including the stiffness matrix, augmented stiffness matrix, displacement vector, and reconstructed force distribution on hand. (**C**) The statistical summary of the force estimation accuracy of three different methods (FEM, CNN, and FEM-NN). (**D**) The relative force error distribution of FEM, CNN, and FEM-NN along force magnitude. (**E**) The quantitative evaluation of the absolute force error distribution of FEM, CNN, and FEM-NN in different force ranges.

We establish the physical model of the outer shell using finite element theory (*43*) taking into account its thin thickness (0.8 mm). As illustrated in Fig. 3Bi and fig. S12, the outer shell is uniformly discretized into a mesh with $N_{e}$ elements and $N_{n}$ nodes, where the mesh size is determined on the marker density to align dimensions. The basic shell element is considered a small rectangular domain, and its length and width are computed on the basis of the geometrical parameters of the outer shell. According to the physical constraints of PhyTac, the nodes in the top layer and the bottom layer are treated as fixed nodes, while the remaining nodes are considered as free nodes with five degrees of freedom (5-DOF), including three displacements and two rotations about the Z axis. Applying the principle of virtual work, the state equation of the element in static equilibrium (*43*) can be presented as follows

$$\iint_{A}\delta {\hat{\varepsilon }}^{T}{\hat{\sigma }}^{T}\mathrm{dA}=\iint_{A}\delta u^{T}t\mathrm{dA}+\delta u^{T}f$$ (1)

where the left side represents the internal virtual strain energy, and the right side represents the external virtual work. Here, u is the virtual displacement vector, $\hat{\varepsilon }$ is the strain vector, $\hat{\sigma }$ is the stress vector, t is the surface load, and f is the force vector.

By substituting the relevant variables to Eq. 1, the state equation can be simplified to $k_{e}\cdot u_{e}=f_{e}$, where $k_{e},u_{e},\text{and}\;f_{e}$ are the element stiffness matrix, element displacement vector, and element force vector (note S2). Then, the relationship between displacements and external force can be obtained as $K\cdot U=F_{p}$ by assembling all elements into the global system, where $K,U,\text{and}\;F_{p}$ are the global stiffness matrix, global displacement vector, and global force vector. K is known from the finite element model (FEM), and U can be derived from the displacement matrix mentioned earlier, allowing for the computation of external forces $F_{p}$.

Figure 3 illustrates the working mechanism of the FEM-NN. Following the principle of the four-node rectangular element of finite element theory (*43*), the displacement/force of a given node i is influenced by the surrounding eight nodes. To compensate for the missing physics (*44*, *45*) of FEM (nonlinear effects, mesh density/quality, order errors, and material errors), we select $nn_{i}={\left[u_{i}\;z_{i}\right]}^{T}$ as the training input and $k_{i}$ as the training output for a cascade neural network (Fig. 3A), where the displacement vector $u_{i}\in {\mathbb{R}}^{9}$ represents the displacements of node i and its eight surrounding nodes, the scalar $z_{i}$ represents the height of the node i in the global coordinate system, and the scalar $k_{i}$ is the augmented local stiffness. Then, the training data were collected experimentally using a 3-DOF automatic test platform comprising three types of grippers, which can make different contact distributions (Materials and Methods and figs. S13 and S14). Because input and output selections of neural network are also based on the principle of FEM, the overall training data are physically meaningful high-quality data. Once the network is trained, consequently, $k_{i}$ (i=1,…,m) can be calculated through the corresponding input ${\left[u_{i}\;z_{i}\right]}^{T}$ by scanning the KOI in the displacement matrix *D*, and the augmented stiffness matrix δK(U) can be obtained as

$$\delta K\left(U\right)=\left[\begin{array}{ccc} k_{1} & \cdots & 0\\ \vdots & \ddots & \vdots \\ 0 & \cdots & k_{m} \end{array}\right]=\left[\begin{array}{ccc} g\left(nn_{1}\right) & \cdots & 0\\ \vdots & \ddots & \vdots \\ 0 & \cdots & g\left(nn_{m}\right) \end{array}\right]$$ (2)

where the g() represents the trained cascade neural network, δK() represents the procedures of circularly scanning the KOI of the displacement matrix and extracting the corresponding input $nn_{i}$ from the displacement matrix, and m is the number of KOI. Last, the external forces F can be reconstructed by adding the FEM part and the augmented part (neural network) (Fig. 3B and note S3)

$$F=\left[\left(K+\delta K(U\right)\right]\cdot U$$ (3)

Benefiting from the $C^{0}$ continuity of shell element in the finite element model, the applied force at any arbitrary position on the outer shell can be theoretically calculated by interpolation.

### Distributed force measurement based on small datasets

We calibrate the FEM-NN model and compare the force reconstruction accuracy of FEM-NN (Fig. 3, C and D) with the physical model–based method (FEM) and convolutional neural network–based method (CNN) using single-point contacts and multiple-point contacts (Materials and Methods and note S5). It indicates that the accuracy of FEM-NN remains remarkably high across the entire force range (0.5 to 25 N) with an average force magnitude error of ~0.11 N and an average relative error of only 2.3%. In comparison, the average relative error by FEM is around 18%, particularly high (49.0%) when the applied force is small (<5 N). The overall accuracy of CNN is approximately 93.0%, which approaches the accuracy of FEM-NN. But note that the dataset of CNN includes 3909 samples (>1 GB), over six times larger than the needed dataset of our FEM-NN (598 samples, 45 KB), and the accuracy of CNN would reduce largely to 48% if using the same small imagesets (fig. S15).

Figure 3E further demonstrates that the quantitative distribution of absolute force errors by FEM-NN is concentrated around 0 N with a small SD (<0.16 N) over the entire force range. This performance is superior to the other two conventional methods, which exhibit large means (>0.35 N) and large SD (>0.28 N).

To better evaluate the performance of our method in real scenarios, we also conduct real hand grip experiments in different grip orientations, positions, or directions, applying forces in a large range from 0.5 to 25 N (fig. S16). The overall accuracy of real hand grip is at the same level as the data from the automated platform (fig. S17), which can validate the generalizability of our FEM-NN model. Furthermore, our device remains robust across different grip orientations and directions, as shown in figs. S18 to S20, due to the working mechanism not being affected by gravity. Then, the accuracy remains stable among different heights of 5 to 110 mm, with only a slight reduction of approximately ~4% at the narrow region near the bottom (105 to 110 mm) (fig. S18). Considering that the middle area (heights of 15 to 95 mm) is the most frequent contact area, the overall error is still within an acceptable error range. The results also clearly reveal that the majority of forces are distributed on fingertips and musculus flexor pollicis brevis, aligning with our intuitive experience in daily life (Fig. 3B, fig. S21, and note S4). Note that the sensing range from 0.5 to 25 N is only for one single point, so if applying force on multiple points, the maximum total force can be up to more than 200 N, and this large range will help our PhyTac applied on many applications, such as sports and VR. Repeated stability test over 1000 cycles was performed, and the PhyTac exhibits no signal drift or fluctuation during the cyclic tests (fig. S22).

It is worth noting that our FEM-NN model requires only a small dataset (just 45 KB) for training. The digital channel allows us to directly extract the location information of the load. Benefited from such high-quality data without interference, the searching space in FEM-NN can be finely constrained within the KOI and its surrounding eight markers’ displacements, as shown in Fig. 3A. In contrast, the searching space of CNN is unconstrained and stochastic over the input images (original images or the entire displacement matrix) with thousands of dimensions. Compared with the conventional method, our approach reduces the number of training parameters by more than 2000 times, from 758.4K (CNN) to 351 (FEM-NN). Consequently, our method requires only minimal computing resources, and the training process can be completed within 1 min (fig. S23), a notable improvement compared to the several hours needed for other image-based deep neural networks.

The introduced physical model establishes a theoretical mechanical framework to connect the marker displacement and force magnitude and provides valuable prior knowledge that incorporates material, geometric, and mechanical properties of PhyTac. In real practice, because of the limitations of mesh density/quality, order errors, material errors, etc., the FEM model is usually with partially missing physics (*44*, *45*), resulting in low accuracy. For this weakness, the FEM-NN model endorses the neural network with the powerful fitting ability, effectively compensating for the missing physics between the finite element model and the real-world model. The results verify that FEM-NN can better model the overall physics and guarantee the reasonability and reliability in force reconstruction. Note that the physical model is a little bit similar but not equal to the pretrain model, due to the stiffness matrix is derived from the mechanical model and does not need a training process.

### Demonstrations for hand force estimation, the virtual world, and robot connection

**Fig. 4.**
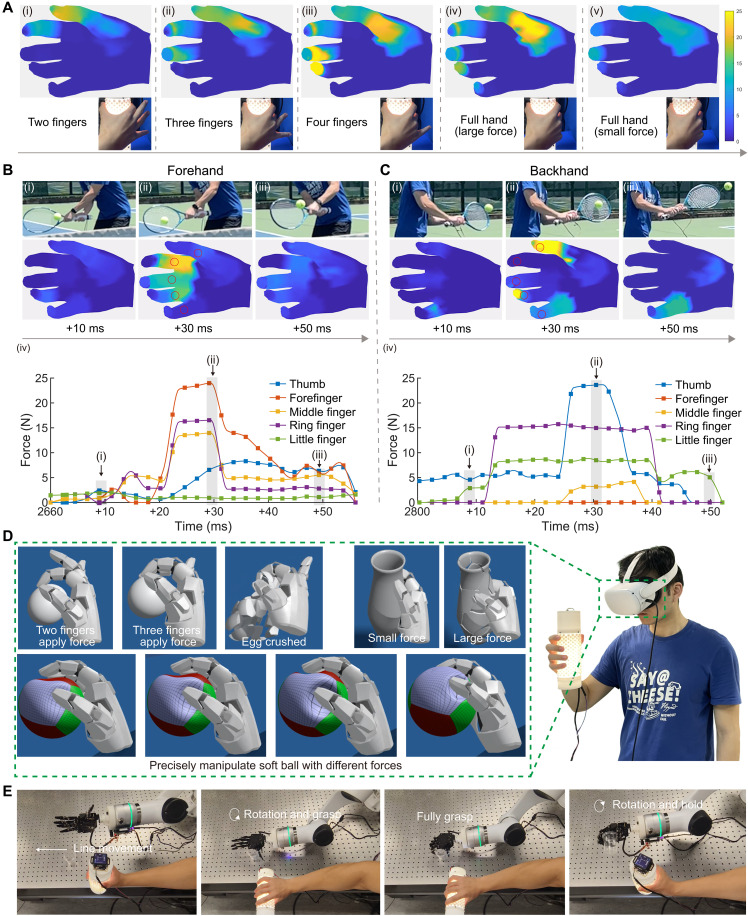
Application demonstrations of PhyTac in multiple finger grasping, dynamic hand grip force measuring in tennis playing, VR interactions, and robot teleoperation. (**A**) Grasping the PhyTac using different numbers of fingers validates the capabilities of PhyTac for hand grip force measurement. (**B**) Force distributions in forehand tennis playing at prestroking [(B), i], stroking [(B), ii], and poststroking [(B), iii]. [(B), iv] Force changes of five points of fingers with time. (**C**) Force distributions in backhand tennis playing at prestroking [(C), i], stroking [(C), ii], and poststroking [(C), iii]. [(C), iv] Force changes of five points of fingers with time. (**D**) Precisely grasp, hold, crush, and deform objects in the virtual world by projecting the force from hand in the real world. (**E**) Using PhyTac to control the robot hand to grasp and move a thin plastic cup without collapsing.

The PhyTac provides a straightforward solution for estimating the strength and distribution of forces generated by hands (*16*), directly benefiting evaluation and treatment of various diseases (stoke, rheumatoid arthritis, etc.). In comparison to other potential solutions like gloves (*17*), our approach stands out for its robustness, ease of use, and unobtrusiveness. As depicted in Fig. 4A and movie S2, it adeptly recognizes dynamic changes in force distribution from all fingers and hands across different gripping postures and strengths. Intuitively, we observe that the largest force concentrates on the fingertips of the thumb when using the thumb and forefinger for grasping (Fig. 4Ai), shifts to the musculus flexor pollicis brevis when using three fingers (Fig. 4Aii), and appears in the middle finger and ring finger when using four fingers (Fig. 4Aiii). In addition, we note that the force distribution from our hand varies with different applied strengths to grasp the object. For instance, the largest force is primarily applied by the musculus flexor pollicis brevis in the case of high strength (Fig. 4Aiv), while the force is mainly contributed by the thumb and fingertips of the other fingers in the case of low strength (Fig. 4Av). These quantitative insights enhance our understanding of hand actions at a high-precision level. Furthermore, the precise grip force distribution contains many rarely decoded information, which suggests the conditions of hand joints and muscles, making it potentially applicable in the evaluation and treatment of various diseases, such as stroke (*16*), and rheumatoid arthritis (*46*).

The precise force production from different regions of the hands forms the foundation for most sports competitions. The advancements of PhyTac in distributed sensing ability and large sensing range position it as a smart sports instrument for quantitatively evaluating such actions. As depicted in Fig. 4 (B and C) and movie S3, the PhyTac, when installed on the tennis racket (fig. S24), can provide real-time dynamic force strength distribution from hands during tennis playing. Figure 4B illustrates that during forehand hitting, the applied force is primarily distributed in the forefinger, middle finger, and ring finger, along with their surrounding parts of the palm. The results in Fig. 4B (ii to iv) show that the largest force (23.9 N) occurs at +30 ms in the region of the forefinger, with the thumb and little finger contributing smaller forces (<10 N) in this process. In contrast, the backhand hitting (Fig. 4C) involves a different force distribution, with the thumb, ring finger, and little finger contributing more force. Specifically, the thumb produces the largest force (23.6 N) to hit the ball, while the ring finger and little finger appear to act as a fulcrum point in rotating the racket (Fig. 4C, ii to iv). Outputting at least two orders of magnitude larger parameters than traditional devices (*47*), our distributed force decoding device offers a scientific tool for deeper understanding of the interaction between plays and rackets, finally improving players’ performance and preventing injuries (*48*).

We also demonstrate that PhyTac can enable precise VR manipulation by projecting force distribution from the real world to the virtual world. While in the real world, we can use different forces to grasp, hold, and crush objects like eggs, replicating these actions in the virtual world has been challenging due to the lack of a distributed force-based interaction interface. PhyTac can accurately transfer the produced force from different fingers to the virtual world, enabling force-induced manipulation, as shown in Fig. 4D and movie S4. The demonstrations illustrate that we can delicately hold an egg using a thumb and forefinger without crushing it or scratch it using three fingers when the total force exceeds the threshold (50 N). Similarly, we demonstrate the scratching of a vase (total force >80 N) and the manipulation of a softball (ranging from 5 to 20 N). Furthermore, leveraging PhyTac’s excellent dexterity in 3D space, it can serve as a teleoperation interface by integrating the inertial measurement unit (IMU) to control the motion of a robotic finger for manipulation that is sensitive to gripping force. As demonstrated in Fig. 4E, we are able to control the robot finger to pick up, hold, and transfer a thin plastic cup without crushing (movie S5). Note that, different from existing methods (table S3), our device can directly control the force to avoid damaging objects, in addition to its lightweight and low cost. These features position PhyTac as a promising device to bridge the gap between the physical world, virtual world, and robots, with potential long-term impact in various fields, including prosthetic robot sensing, safety control, precise remote control of surgical robots, and human-robot interaction.

## DISCUSSION

This research yields a digital channel–enabled distributed force decoding strategy and a robust tactile interactive system capable of reconstructing dynamic spatial-temporal hand force. In particular, the introduction of a digital channel to force decoding can extract the location of force from the coupled analogy signal, offering physically meaningful high quality training data and easing force reconstruction processing. The bioinspired spiral arrangement obtains the largest maker density on record in parallel-type sensing surfaces and overcomes the marker overlap issues in existing marker-based devices. Furthermore, the FEM-NN architecture can reconstruct the force with an ultrasmall dataset, not only addressing the arbitrary extrapolation and massive dataset challenges in force mapping but also paving notable concepts for the development of soft tactile sensors and AI algorithms. Two prototypes with different sizes validate not only the effectiveness of our strategies but also the excellent robustness and high accuracy. It is worth noting that, while the diameters of our prototypes are 40 and 60 mm, the design is easily adjustable to fit hands of various sizes. In addition to the cylinder geometry, our design framework is also compatible with other shapes, including planes, cones, cuboids, hemispheres, and curved surfaces (fig. S25). In addition to the camera-based tactile sensors, the concept of digital channel could also be applied to other types of distributed soft tactile sensors to overcome interference problems and accurately locate complex stimuli, especially in high-density arrays. While adding a layer of digital channel may complicate the fabrication process, it is also a viable option for researchers to improve distributed force accuracy. This work stands out from previous approaches (table S2) in terms of small datasets, large sensing range, large sensing area, and high accuracy, laying the groundwork for broader applications in VR, augmented reality, mixed reality, health care, sports, and robotics.

## MATERIALS AND METHODS

### Materials and fabrication of the PhyTac

A schematic illustration of the common fabrication process is depicted in fig. S26. The outer shell of PhyTac was fabricated by 3D printing, using a photosensitive resin material with excellent toughness (3D printer, lite600hd, Ningbo Zhechuang Technologies Co. Ltd., China; material, C-UV 9400E, Wenext Technology Co. Ltd., China). The markers of the outer shell were manually colored using blue and green mark pen (bluemark pen: grasp 64; green mark pen: grasp 46, Wenzhou Jinma Stationery Manufacturing Co. Ltd., China). We selected thermoplastic polyurethane (TPU) elastomer (Hei-Cast 8400N, H&K Ltd., Japan) as the mouldable material for the soft threshold switch. Parts A and C of the TPU were combined in a weight ratio of 1:5, followed by stirring the mixture for 2 min. A vacuum pump was used to remove bubbles from the mixture for 5 min. Subsequently, the mixture of parts A and C was added into part B in a weight ratio of 6:1, and then the mixture was stirred for 30 s and bubbles were removed for an additional minute. All these processes were conducted at 25° to 35°C. The mixture was then injected into a prepared silicon mould and cured at 65°C for 1 hour. After demoulding, the tips of threshold switches were colored with a red mark pen (grasp 15). Threshold switches were then integrated into the outer shell using an adhesive (Loctite 406, Henkel AG & Co. KGaA, Germany). The COB light strip (Shenzhen Greethink Electronic Co. Ltd., China), polarizing films (Shenzhen Yijia Photo Products Co. Ltd., China), and 3D-printed light source skeleton were integrated using double faced adhesive tape. The connectors were fabricated using 3D printing from lite600hd. The camera used in tennis playing (KS1.3A142-GS, Shenzhen Jingcent Photo Products Co. Ltd., China) has a resolution of 640 × 480 and a frame rate of 480 fps, and the camera used for other cases (HF500, Shenzhen JRWT Electronic Co. Ltd., China) has a resolution of 2592 × 1944 and a frame rate of 30 fps. The connectors, camera, outer shell, and light source were assembled by screws.

### Test platform for PhyTac calibration and data collection

Two test platforms are established to collect training data and validation data. The 3-DOF automatic test platform, used for PhyTac calibration and training data collection, is depicted in figs. S13 and S14. One DOF controls the Cartesian movement of the PhyTac along the *Z* axis using a linear screw actuator with a precision of 0.03 mm (Sheng Starr Transmission Technology Co. Ltd., China), and one DOF controls the rotation angle of PhyTac around *Z* axis using a closed-loop stepper motor (Dekesi Motor) with a precision of 0.0036°, and the final DOF controls the grasping depth of graspers using a bidirectional linear screw actuator with a precision of 0.06 mm. The graspers, indenters, and connectors were fabricated using 3D printing. For single-point contacts, standard force sensors (HZC-MS1, Cy Sensors, China) with a force precision of 0.03 N were integrated into the grippers to collect ground truth force data. For multiple-point contacts, another nine standard force sensors (DYHW-108, Dayang Sensors, China) were integrated into two types of grippers to collect truth data, as shown in fig. S14. Considering that the PhyTac exhibits 3D axial symmetry, data collection is only required for markers between two adjacent columns. This platform can automatically traverse the selected columns by applying different force values under quasi-static conditions and can automatically and simultaneously record the ground truth force distribution and the corresponding dynamic image from the camera. Furthermore, we also established a validation platform to collect data of real hand grip (fig. S16). This platform includes nine standard force sensors (DYHW-108), which are distributed across five fingers of a glove. Wearing this glove, we gripped the PhyTac in different grip orientations, positions, and directions to collect data and verify its robustness and generalizability.

### Calibration of the FEM model and the cascade neural network

The parameters of FEM (Young’s modulus, Poisson’s ratio, etc.) of the outer shell were calibrated on the basis of the collected force data. This calibration could be simplified as a minimization problem, expressed as follows

$$\underset{p}{\text{min}}\left[\sum_{i=1,\ldots ,n}\left\|F_{\text{gt}i}-K\left(p\right)\cdot U_{i}\right\|\right]$$ (4)

In this equation, p represents the parameters, $F_{\mathrm{gt}}$ the ground truth force, and n the number of samples. By minimizing the difference between the experimentally ground truth and FEM estimation, the calibrated FEM model could be used to estimate the force distribution (fig. S27). As for the cascade neural network, the collected data include 598 samples with random external force values, among which around 48% data are collected by multiple-point contacts and the other is collected by single-point contacts. The dataset is split into training, validation, and test sets with a ratio of 8.5:0.5:1. The neural network is composed of three layers (except for the input layer), with 10 hidden units in each of the two hidden layers, and the activation function of each hidden neuron is a linear unit.

### Virtual scenarios setup for application of VR interactions

The 3D VR environment and objects were designed with Unity3D (Unity Technologies) on a laptop computer and were projected into the VR headset (Oculus Quest 2, Meta) through Oculus Link. In Unity3D, the interaction between the PhyTac and VR environment was facilitated through the Transmission Control Protocol (TCP). The bending of virtual finger and applied force were controlled by the distributed force of individual fingers on the PhyTac, while the location and orientation of the virtual hand were tracked by the hand-tracking function of the Oculus. Unity3D used sphere and capsule collision on a hand model to detect the object contact via Unity3D script execution. When the virtual hand was not in contact with virtual objects, the bending of virtual fingers was independently controlled by the real applied force of each finger. When the virtual hand contacted an object, the distribution of applied force in real world was directly projected onto the virtual object, thereby mimicking precise manipulations in reality. As for the virtual objects, the egg and the vase could be crushed when the total force reached 50 and 80 N respectively, and the deformation of the soft ball is achieved through mesh deformation when the distributed force is applied.

### Robot teleoperation setup for application of human-robot interactions

The robotic hand (Hiwonder Technology Co. Ltd., China) was integrated with the robotic arm (E03-Pro, Han’s Robot Co. Ltd., China) using a 3D-printed connector. The bending of the robotic hand’s fingers was controlled by the force distribution on the PhyTac via the serial port communication. Concurrently, the position and orientation of the robotic arm’s end were open-loop controlled by the IMU (IM948, Chenyi Electronic Co. Ltd., China), which is integrated into the top of the PhyTac. The computer first collects the IMU results via Bluetooth and then gives commands to the robotic arm via TCP after information processing.

## Supplementary Materials

### The PDF file includes:

Supplementary Text Figs. S1 to S27 Tables S1 to S3 Legends for movies S1 to S5 References
