# Supplementary material for: Digital channel–enabled distributed force decoding via small datasets for hand-centric interactions
Source: Sci Adv. 2025 Jan 22;11(4):eadt2641. doi: 10.1126/sciadv.adt2641 (PMC11753382; doi:10.1126/sciadv.adt2641)
Supplement: Supplementary file 1 — Supplementary Text Figs. S1 to S27 Tables S1 to S3 Legends for movies S1 to S5 References [file sciadv.adt2641_sm.pdf]

Science Advances
AAAS

Supplementary Materials for

Digital channel–enabled distributed force decoding via small datasets for hand-centric interactions

Yifeng Tang *et al.*

Corresponding author: Yajing Shen, eeyajing@ust.hk

Sci. Adv. **11**, eadt2641 (2025)
DOI: 10.1126/sciadv.adt2641

The PDF file includes:

Supplementary Text
Figs. S1 to S27
Tables S1 to S3
Legends for movies S1 to S5
References

Other Supplementary Material for this manuscript includes the following:

Movies S1 to S5

Supplementary Text

Note S1 Mapping relationship between image coordinate and WCS

After obtaining the image coordinates of all markers, as shown in Supplementary Figure S2, the next key step is converting the image coordinates to world (global) coordinates. Supplementary Figure S3 illustrates the geometrical relationship between two coordinate systems. The Z-axis of WCS aligns with the optical axes of the camera. In image coordinate system, a point q can be described by $q = [x, y]^T \in \mathbb{R}^2$, and the image center o can be described as $o = [x_o, y_o]^T \in \mathbb{R}^2$. In the WCS, the point q_b and q_r can be described as $q_b = [x_b, x_b, x_b]^T \in \mathbb{R}^3, q_r = [x_r, x_r, x_r]^T \in \mathbb{R}^3$, respectively. The projective equation can be obtained based on the geometrical relationships:

$$\begin{bmatrix} x_b \\ y_b \end{bmatrix} = \left(1 + \frac{Z_1 + b}{f}\right) \begin{bmatrix} x \\ y \end{bmatrix}$$

$$z_b = Z_2 - Z_1 - b$$

where $x = u - x_0, y = v - y_0$, f is the focal length of the camera in pixels, Z_1 is the distance between image plane and the nearest marker in Z-axis, Z_2 is the distance between image plane and the origin of WCS in Z-axis, and b is the distance between two adjacent markers in Z-axis. The (u, v) are pixel coordinates of point q in the image plane. Finally, the reconstructed coordinates of the outer shell q_r can be determined to be

$$x_r = x_b + h \cdot cos\varphi$$

$$y_r = y_b + h \cdot sin\varphi$$

$$z_r = z_b$$

where $\varphi = \text{arctan2}(y_b, x_b)$, h is the height of the corresponding receptor, which is known in advance. By cycling all markers by the above mapping relationship, the 3-D shape of the outer shell can be reconstructed as shown in Figure S3.

Note S2 Physical model between the node displacement and the force magnitude

The PhyTac is discretized to a four-node rectangular shell-based flat shell model. According to Reissner-Mindlin flat shell theory, the coordinates of a node O in the local coordinate system can be expressed as

$$\begin{aligned} u'(x',y',z') &= u'_0(x',y') - z'\theta_{0x'}(x',y') \\ v'(x',y',z') &= v'_0(x',y') - z'\theta_{0y'}(x',y') \\ w'(x',y',z') &= w'_0(x',y') \end{aligned} \tag{7}$$

where u'_0, v'_0 and w'_0 are the displacements of node O along the local directions x', y' and z', respectively; $\theta_{0x'}$ and $\theta_{0y'}$ are the rotation angles in the local planes $x'z'$ and $y'z'$, respectively; the local displacement vector of node O is $\mathbf{d'_0} = \left[u'_0,\ v'_0,\ w'_0\ ,\ \theta_{0x'},\ \theta_{0y'}\right]^T$.

Therefore, the displacement field within a rectangular element can be expressed by

$$\mathbf{u} = \left[u', v', w', \theta_{x'}, \theta_{y'}\right]^T = \sum_{i=1}^{4} \mathbf{N_i d'_i} \tag{8}$$

where $\mathbf{N_i} = \mathrm{diag}(N_\mathrm{i}, N_\mathrm{i}, N_\mathrm{i}, N_\mathrm{i}, N_\mathrm{i})$ represents the matrix of shape functions, $N_i = \frac{1}{4}(1 + \xi_i\xi)(1 + \eta_i\eta)$, with $\xi, \eta \in [-1\ 1]$, $\xi = \frac{x'}{a}, \eta = \frac{y'}{b}$, $\xi_{1,4} = -1, \eta_{1,2} = -1, \xi_{2,3} = 1, \eta_{3,4} = 1$. The relevant strains $\hat{\boldsymbol{\varepsilon}}$ and stresses $\hat{\boldsymbol{\sigma}}$ can be written as follows:

$$\hat{\boldsymbol{\varepsilon}} = \begin{Bmatrix} \boldsymbol{\varepsilon}'_\mathrm{m} \\ \boldsymbol{\varepsilon}'_\mathrm{b} \\ \boldsymbol{\varepsilon}'_\mathrm{s} \end{Bmatrix} = \begin{Bmatrix} \frac{\partial u'}{\partial x'} \\ \frac{\partial v'}{\partial y'} \\ \frac{\partial u'}{\partial y'} + \frac{\partial v'}{\partial x'} \\ \frac{\partial \theta_{x'}}{\partial x'} \\ \frac{\partial \theta_{y'}}{\partial y'} \\ \frac{\partial \theta_{x'}}{\partial y'} + \frac{\partial \theta_{y'}}{\partial x'} \\ \frac{\partial w'}{\partial x'} - \theta_{x'} \\ \frac{\partial w'}{\partial y'} - \theta_{y'} \end{Bmatrix} = \sum_{\boldsymbol{i=1}}^{\boldsymbol{4}} \begin{Bmatrix} \frac{\partial N_i}{\partial x'} u'_i \\ \frac{\partial N_i}{\partial y'} v'_i \\ \frac{\partial N_i}{\partial y'} u'_i + \frac{\partial N_i}{\partial x'} v'_i \\ \frac{\partial N_i}{\partial x'} \theta_{x'_i} \\ \frac{\partial N_i}{\partial y'} \theta_{y'_i} \\ \frac{\partial N_i}{\partial y'} \theta_{x'_i} + \frac{\partial N_i}{\partial x'} \theta_{y'_i} \\ \frac{\partial N_i}{\partial x'} w'_i - N_i \theta_{x'_i} \\ \frac{\partial N_i}{\partial y'} w'_i - N_i \theta_{y'_i} \end{Bmatrix} = \sum_{\boldsymbol{i=1}}^{\boldsymbol{4}} \begin{Bmatrix} \mathrm{B}'_{m_i} \\ \mathrm{B}'_{b_i} \\ \mathrm{B}'_{s_i} \end{Bmatrix} \mathbf{u}'_{\boldsymbol{i}} = \sum_{\boldsymbol{i=1}}^{\boldsymbol{4}} \mathbf{B}'_{\boldsymbol{i}} \mathbf{u}'_{\boldsymbol{i}} \tag{9}$$

$$\hat{\boldsymbol{\sigma}} = \begin{Bmatrix} \boldsymbol{\sigma}'_\mathrm{m} \\ \boldsymbol{\sigma}'_\mathrm{b} \\ \boldsymbol{\sigma}'_\mathrm{s} \end{Bmatrix} = \begin{bmatrix} \mathrm{D}_m & 0 & 0 \\ 0 & \mathrm{D}_b & 0 \\ 0 & 0 & \mathrm{D}_s \end{bmatrix} \begin{bmatrix} \boldsymbol{\varepsilon}'_\mathrm{m} \\ \boldsymbol{\varepsilon}'_\mathrm{b} \\ \boldsymbol{\varepsilon}'_\mathrm{s} \end{bmatrix} = \mathbf{D}\hat{\boldsymbol{\varepsilon}} \tag{10}$$

where $\mathrm{B}'_{m_i}, \mathrm{B}'_{b_i}$ and B'_{s_i} are the membrane, bending and transvers shear strain matrices, respectively, given by

$$\mathrm{B}'_{m_i} = \begin{bmatrix} \frac{\partial N_i}{\partial x'} & 0 & 0 & 0 & 0 \\ 0 & \frac{\partial N_i}{\partial y'} & 0 & 0 & 0 \\ \frac{\partial N_i}{\partial y'} & \frac{\partial N_i}{\partial x'} & 0 & 0 & 0 \end{bmatrix}, \mathrm{B}'_{b_i} = \begin{bmatrix} 0 & 0 & 0 & \frac{\partial N_i}{\partial x'} & 0 \\ 0 & 0 & 0 & 0 & \frac{\partial N_i}{\partial y'} \\ 0 & 0 & 0 & \frac{\partial N_i}{\partial y'} & \frac{\partial N_i}{\partial x'} \end{bmatrix}, \mathrm{B}'_{s_i} = \begin{bmatrix} 0 & 0 & \frac{\partial N_i}{\partial x'} & -N_i & 0 \\ 0 & 0 & \frac{\partial N_i}{\partial y'} & 0 & -N_i \end{bmatrix}$$

The constitutive matrices are given by

$$\mathrm{D}_m = \frac{Et}{1-v^2}\begin{bmatrix} 1 & v & 0 \\ v & 1 & 0 \\ 0 & 0 & \frac{1-v}{2} \end{bmatrix}, \mathrm{D}_b = \frac{t^2}{12}\mathrm{D}_m, \mathrm{D}_s = \frac{\kappa Et}{2(1+v)}\begin{bmatrix} 1 & 0 \\ 0 & 1 \end{bmatrix}$$

where E, v, and t are respectively the elastic modulus, Poisson's ratio, and shell thickness, κ is the shear correction factor and equals 5/6 for isotropic materials. By applying the principle of virtual work (equation 1) and substituting the constitutive equation (10) into the equation (1), we have

$$\iint_A \delta\hat{\boldsymbol{\varepsilon}}^T D\hat{\boldsymbol{\varepsilon}}\, dA - \iint_A \delta\boldsymbol{u}^T \boldsymbol{t} dA = \delta\boldsymbol{u}^T f \tag{11}$$

Substituting the equation (8) and (9) into equation (11), the equilibrium equations for element are given as

$$k'_e \cdot u'_e = f'_e$$

where f'_e represents the local force vector, combining the face load and node load, and the local stiffness matrix k'_e can be obtained by

$$\begin{aligned} k'_e &= \iint_A \mathbf{B}'^{T}_i\mathbf{D}\mathbf{B}'_{\mathrm{j}} dA = ab\int_{-1}^{1}\int_{-1}^{1} \mathbf{B}'^{T}_i\mathbf{D}\mathbf{B}'_{\mathrm{j}} d\xi d\eta \\ &= ab\int_{-1}^{1}\int_{-1}^{1} \begin{bmatrix} \mathrm{B}'_{m_i} \\ \mathrm{B}'_{b_i} \\ \mathrm{B}'_{s_i} \end{bmatrix}^T \begin{bmatrix} \mathrm{D}_m & 0 & 0 \\ 0 & \mathrm{D}_b & 0 \\ 0 & 0 & \mathrm{D}_s \end{bmatrix} \begin{bmatrix} \mathrm{B}'_{m_i} \\ \mathrm{B}'_{b_i} \\ \mathrm{B}'_{s_i} \end{bmatrix} d\xi d\eta \\ &= k'_m + k'_b + k'_s \end{aligned} \tag{12}$$

where

$$k'_m = ab\int_{-1}^{1}\int_{-1}^{1} \mathrm{B}'^{\mathrm{T}}_{m_i}\mathrm{D}_m\mathrm{B}'_{m_j} d\xi d\eta$$

$$k'_b = ab\int_{-1}^{1}\int_{-1}^{1} \mathrm{B}'^{\mathrm{T}}_{b_i}\mathrm{D}_b\mathrm{B}'_{b_j} d\xi d\eta$$

$$k'_s = ab\int_{-1}^{1}\int_{-1}^{1} \mathrm{B}'^{\mathrm{T}}_{s_i}\mathrm{D}_s\mathrm{B}'_{s_j} d\xi d\eta$$

Then, the global system can be described as

$$\begin{aligned} u_e &= L_i(\phi) \cdot u'_e \\ f_e &= L_i(\phi) \cdot f'_e \\ k_e &= L_i(\phi) \cdot k'_e \cdot L_i^T(\phi) \end{aligned} \tag{13}$$

where the transformation matrices $L_i(\phi)$ is formulated as

$$L_i(\phi) = \begin{bmatrix} 0 & -sin\phi & cos\phi & 0 & 0 \\ 0 & cos\phi & sin\phi & 0 & 0 \\ -1 & 0 & 0 & 0 & 0 \\ 0 & 0 & 0 & sin\phi & 0 \\ 0 & 0 & 0 & -cos\phi & 0 \\ 0 & 0 & 0 & 0 & -1 \end{bmatrix} \tag{14}$$

the angle ϕ of an element is the angle between the global x axis and normal vector of this element. Finally, the global element state equation can be described as equation (2). By assembling all elements to the global system, the state equation can be obtained as equation (3).

Note S3 One example to describe the working principle of FEM-NN

To clearly describe the FEM-NN, we take one example (with nine nodes and four elements) as shown in Fig. S1.

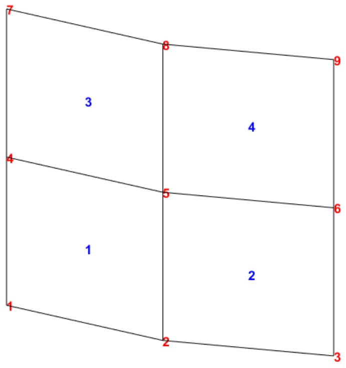

Fig. S1 A curved shell with 9 nodes and 4 elements.

The displacement field of each node (such as node 5 in the center) can be expressed by

$$\mathbf{d_0'} = \left[u_0',\ v_0',\ w_0'\ ,\ \theta_{0x'},\ \theta_{0y'}\right]^T.$$

where u_0', v_0' and w_0' are the displacements of node O along the local directions x', y' and z', respectively; $\theta_{0x'}$ and $\theta_{0y'}$ are the rotation angles in the local planes $x'z'$ and $y'z'$, respectively. And for a rectangular element (such as element 1 in the left bottom), its displacement field can also be expressed by

$$\mathbf{u} = \left[u', v', w', \theta_{x'}, \theta_{y'}\right]^T = \sum_{i=1}^{4} \mathbf{N_i d_i'}$$

which means the displacement of an arbitrary point in the element can be interpolated by the four nodes (nodes 1, 2, 4, 5). Finally, based on the principle of virtual work (equation 1) and materials mechanics (equation 9-10), the equilibrium equations for this element in the local system are given as

$$k_e' \cdot u_e' = f_e'$$

And the local stiffness matrix k_e' can be obtained by

$$k_e' = \iint_A \mathbf{B}_i'^{\boldsymbol{T}} \mathbf{D} \mathbf{B}_\mathbf{j}' dA = ab \int_{-1}^{1} \int_{-1}^{1} \mathbf{B}_i'^{\boldsymbol{T}} \mathbf{D} \mathbf{B}_\mathbf{j}' d\xi d\eta$$

$$= ab \int_{-1}^{1} \int_{-1}^{1} \begin{bmatrix} \mathrm{B}_{m_i}' \\ \mathrm{B}_{m_i}' \\ \mathrm{B}_{m_i}' \end{bmatrix}^T \begin{bmatrix} \mathrm{D}_m & 0 & 0 \\ 0 & \mathrm{D}_b & 0 \\ 0 & 0 & \mathrm{D}_s \end{bmatrix} \begin{bmatrix} \mathrm{B}_{m_i}' \\ \mathrm{B}_{m_i}' \\ \mathrm{B}_{m_i}' \end{bmatrix} d\xi d\eta$$

$$= k_m' + k_b' + k_s'$$

The equilibrium equations in the global system can be obtained by coordinate transformation (equation 13)

$$k_e \cdot u_e = \quad .$$

The element stiffness matrix has a dimension of 24 × 24 (4 nodes with 6 degrees of freedom). For one node, the sub-matrix (6 × 6) can be shown as follows,

$$k_{e11} = \begin{bmatrix} 2.0248 & -0.7630 & -0.6287 & -0.0010 & 0.0018 & -0.0018 \\ -0.7630 & 3.0445 & 1.1763 & -0.0005 & 0.0010 & -0.0009 \\ -0.6287 & 1.1763 & 4.0321 & 0 & 0 & 0 \\ -0.0010 & -0.0005 & 0 & 1.34e^{-6} & -2.51e^{-6} & 2.57e^{-8} \\ 0.0018 & 0.0010 & 0 & -2.51e^{-6} & 4.69e^{-6} & -4.80e^{-8} \\ -0.0018 & -0.0009 & 0 & 2.57e^{-8} & -4.80e^{-8} & 6.01e^{-6} \end{bmatrix}$$

$$\begin{matrix} \uparrow & \uparrow & \uparrow & \uparrow & \uparrow & \uparrow \\ u_1 & v_1 & w_1 & \theta_{x1} & \theta_{y1} & \theta_{z1} \end{matrix}$$

Because the rotation and moment items are very small and can be neglected, for easy presentation, we can extract displacement related items and simplify the element stiffness matrix (element 1) as shown in Fig. S2.

Nonzero submatrix

$$k_e = \begin{bmatrix} 2.0248 & -0.7630 & -0.6287 & -0.5544 & 0.6459 & 0.0484 & -1.0124 & 0.3815 & 0.6287 & -0.4580 & -0.2644 & -0.0484 \\ -0.7630 & 3.0445 & 1.1763 & 0.6459 & -1.4176 & -0.0905 & 0.3815 & -1.5222 & -1.1763 & -0.2644 & -0.1046 & 0.0905 \\ -0.6287 & 1.1763 & 4.0321 & -0.0484 & 0.0905 & 0.7949 & 0.6287 & -1.1763 & -2.0160 & 0.0484 & -0.0905 & -2.8109 \\ -0.5544 & 0.6459 & -0.0484 & 2.0248 & -0.7630 & 0.6287 & -0.4580 & -0.2644 & 0.0484 & -1.0124 & 0.3815 & -0.6287 \\ 0.6459 & -1.4176 & 0.0905 & -0.7630 & 3.0445 & -1.1763 & -0.2644 & -0.1046 & -0.0905 & 0.3815 & -1.5222 & 1.1763 \\ 0.0484 & -0.0905 & 0.7949 & 0.6287 & -1.1763 & 4.0321 & -0.0484 & 0.0905 & -2.8109 & -0.6287 & 1.1763 & -2.0160 \\ -1.0124 & 0.3815 & 0.6287 & -0.4580 & -0.2644 & -0.0484 & 2.0248 & -0.7630 & -0.6287 & -0.5544 & 0.6459 & 0.0484 \\ 0.3815 & -1.5222 & -1.1763 & -0.2644 & -0.1046 & 0.0905 & -0.7630 & 3.0445 & 1.1763 & 0.6459 & -1.4176 & -0.0905 \\ 0.6287 & -1.1763 & -2.0160 & 0.0484 & -0.0905 & -2.8109 & -0.6287 & 1.1763 & 4.0321 & -0.0484 & 0.0905 & 0.7949 \\ -0.4580 & -0.2644 & 0.0484 & -1.0124 & 0.3815 & -0.6287 & -0.5544 & 0.6459 & -0.0484 & 2.0248 & -0.7630 & 0.6287 \\ -0.2644 & -0.1046 & -0.0905 & 0.3815 & -1.5222 & 1.1763 & 0.6459 & -1.4176 & 0.0905 & -0.7630 & 3.0445 & -1.1763 \\ -0.0484 & 0.0905 & -2.8109 & -0.6287 & 1.1763 & -2.0160 & 0.0484 & -0.0905 & 0.7949 & 0.6287 & -1.1763 & 4.0321 \end{bmatrix}$$

$$\begin{matrix} \uparrow & \uparrow & \uparrow & \uparrow & \uparrow & \uparrow & \uparrow & \uparrow & \uparrow & \uparrow & \uparrow & \uparrow \\ u_1 & v_1 & w_1 & u_2 & v_2 & w_2 & u_4 & v_4 & w_4 & u_5 & v_5 & w_5 \end{matrix}$$

node1 node2 node4 node5

Figure S2 stiffness matrix of element 1 with nodes 1,2,4 and 5

Similar to element 1, the stiffness matrix of elements 2-4 can also be computed. Then, the global matrix can be assembled by elements 1-4, as illustrated in Fig.S3, where the nonzero submatrix is a part of the element stiffness matrix, as shown in Fig. S2.

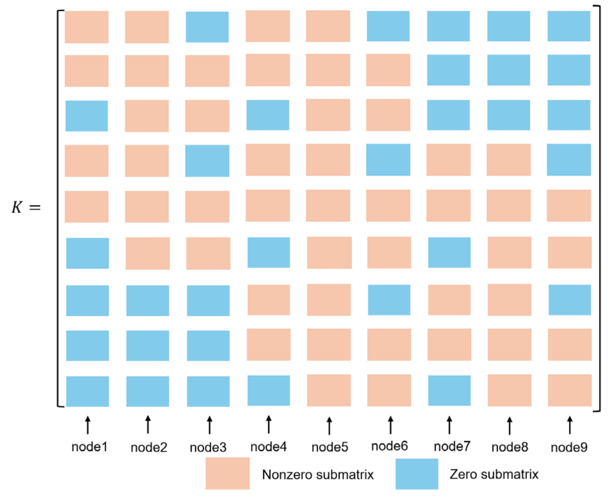

Figure S3 Global stiffness matrix of the example with 4 elements and 9 nodes

Finally, the external force can be calculated by

$$K \cdot \boldsymbol{U} = \boldsymbol{F_p}$$

where U is the displacement vector of the 9 nodes and F_p is the external force vector. For the physical model based force reconstruction in the manuscript, we use the above process to unravel cylindrically connected nodal array into rectangular grids or stiffness matrix, because we can see that the global stiffness matrix has the connection information among different nodes. For example, node 3 only has nonzero submatrices in the places of nodes 2,3,5,6, which corresponds to the Figure S3.

Following the principle of the four-node rectangular element of finite element theory, we can also know that the displacement/force of one node is influenced by the surrounding eight nodes (like node 5 in the example). Therefore, we select the displacements of the surrounding eight nodes and its height as the training input, and its augmented local stiffness as training output to compensate the modelling error of FEM. Because input and output selections of neural network are also based on the principle of FEM, the overall training data are physically meaningful high-quality data, resulting in small dataset can also have great accuracy. The FEM-NN based force reconstruction can be finally obtained by adding the linear part (physical model) and the augmented part (neural network):

$$\boldsymbol{F} = \left(K + \delta K(\boldsymbol{U})\right) \cdot \boldsymbol{U}$$

The key nodes of interest (KOI), obtained by threshold switches, is the index numbers of activated nodes:

$$KOI = \{r_i | i = 1, \dots, m\}$$

where r_i *is* the index of key node and m is the number of key nodes. For example, if nodes 2 and 5 are activated, we know that $KOI = \{2,5\}$, so only the corresponding part of FEM and neural network will be calculated as shown in Fig. S4.

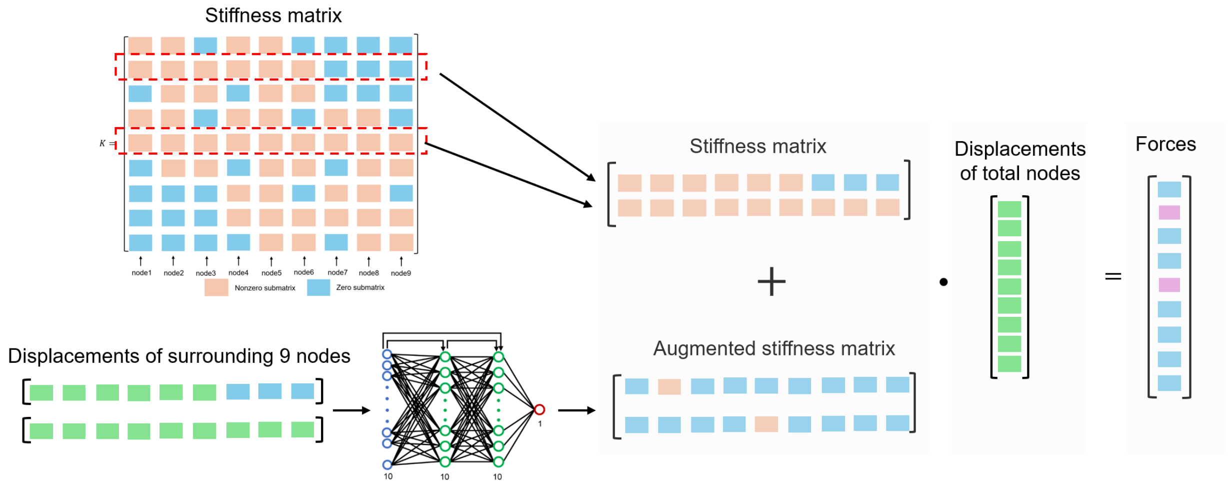

Figure S4 Using KOI to extract required information

Note S4 Projecting force distribution from PhyTac to hand

As shown in Figure S21a, the original distributed force map, obtained from FEM-NN algorithm, is in a 3D format. By unwrapping the 3D map along the circumferential direction, the force distribution can also be represented in a 2D format. However, understanding the relationship between original force formats and hand can be challenging. To easier understand the obtained force map, we directly project the original force distribution on PhyTac to the hand model, and the overall procedures are shown in Figure S21. First, the outer surface of PhyTac is divided into seven regions in the original state, including forefinger, middle finger, ring finger, little finger, thumb, musculus flexor pollicis brevis and palm. Once threshold switches in one region are activated, we can determine which finger or part of hand is applying force. By contrast, if all threshold switches in this region are not activated, we can infer that this finger of part of hand is not applying force. At the same time, the 3D hand model can also be divided into the same seven regions. Then, in every region, the force distribution can be easily projected from PhyTac to hand model point by point. After projecting distributed force of all regions, the hand-based force distribution can be obtained as shown in Figure S21d, which can clearly indicate which part of hand applies more force and is also friendly to broad readers in different fields.

Note S5 Convolutional neural network (CNN) based force reconstruction details

Traditional Convolutional neural network (CNN)-based force reconstruction methods aim to build the relationship between surface deformation and the force map, so they use raw images or image processing-based marker displacement as network input and total force/ distributed force map/ force direction as output. Here, for comparison, we select force map as output for a commonly used DNN architecture ResNet-18 but with customized modifications. The size of input image is 97×125, which is linearly up-sampled from the marker displacement matrix obtained from image processing; the number of channels is three, making it compatible with the ResNet-18. For force map reconstruction, we use a parameter-pretrained ResNet-18 and modify the architecture, using only two ResNet blocks instead of four, and replace the fully connected output by following layers:

1. A convolution layer from 128 to 64 channels.
   Conv2d (128, 64, kernel_size=(3, 3), stride=(1, 1), padding=same)
2. A ReLU layer.
3. A convolution layer to the force map (25 × 32).
   Conv2d (64, 1, kernel_size=(3, 3), stride=(1, 1), padding=same)
4. Regression output layer with a loss function of mean-squared-error.

The network comprises a total of 758.4 thousand parameters. The training data is processed from raw images with 3909 samples.

Note S6 Dynamic image processing and force reconstruction algorithm

The overall process for image processing and force reconstruction proposed in this work is summarized as Algorithm 1 as follows.

Algorithm 1: digital channel enabled dynamic force reconstruction algorithm

```
Input: C (camera), K (stiffness matrix), Neural network, S (stop)
im1 = GetImage(C)
Coor1 = ImageSeg(im1)
i = 1
while i ≤ Inf do
   im2 = GetImage(C)
   Coor2 = ImageSeg (im2)
   DispMat = Coor2 – Coor1
   for j = 1 to Length(Coor2) do
     if j within KOI then
          [U(j), Height(j)] ← DispMat
          DispVec(j) ← U(j)
         δK(j) = NeuralNetwork(DispVec(j), Height(j))
         F(j) = (K(j) + δK(j)) · U(j)
     else
        F(j) = 0
     end if
  end for
  Fd(i) = F
  S = Detect(Stop)
  if S = 1 then
     Break while
  else
     i=i+1
  end if
end while
for n=1 to Length(Fd) do
  Fh(n) = Map2Hand(Fd(n))
end for
Output: Fh (spatio-temporal force distribution on hand)

Function Coor = ImageSeg(im) (processing image to marker coordinates)
     [R, G, B] = ImSeg(im)
     KOI = KeyNode(R)
     p = Combine(G, B)
     gp = Kmedoids(p)
     gpi = Interp(gp)
     Coor = CoorMap(gpi)
end Function
```

The image processing is shown in Supplementary Fig. S9-11, and the force construction process is shown in Fig. 3B. These procedures are run on a desktop PC with an i7-12700H processor at 2.30 GHz and 16 GB of RAM. The algorithm was effectively performed in real-time at about 12 Hz, with time to image-processing ~ 0.06 s and force reconstruction ~ 0.025 s in MATLAB scripts. Regarding the computation speed of FEM-NN, as we have obtained displacement ***U***, the total computation is just a standard matrix multiplication rather than inverse matrix K^{-1} calculation. For the same mesh size (with 768 grids), the computation time is approximately 0.009 s, almost 11 times faster than the process of calculating inverse matrix K^{-1}(~0.1s), as shown in equation (3). The processing frequency can be further improved by using high-performance GPU and algorithm optimization.

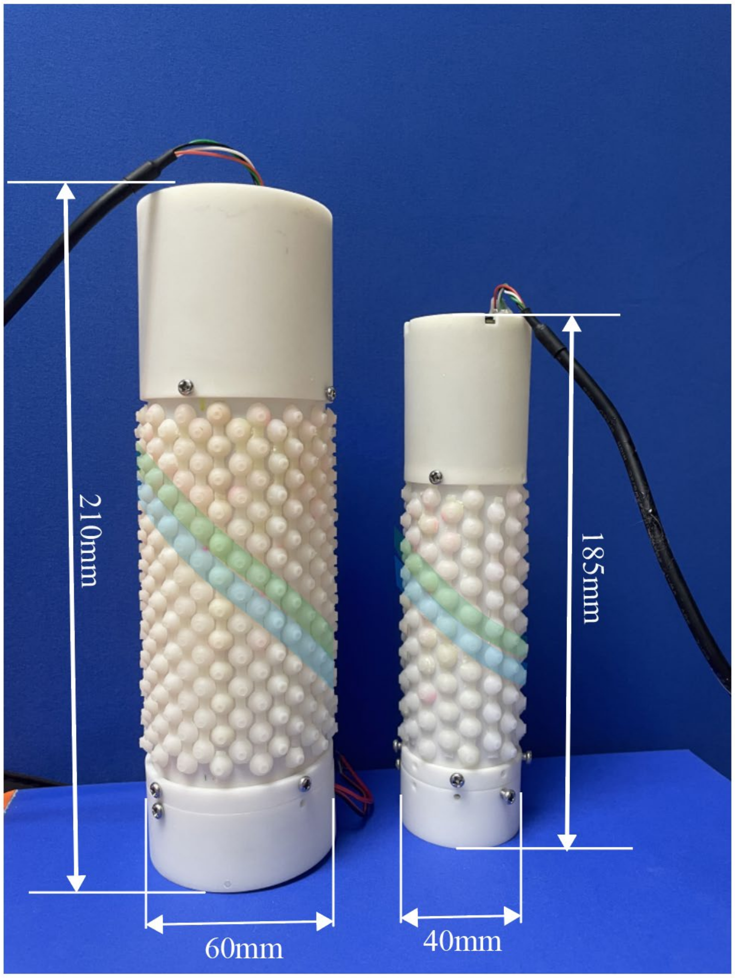

Figure S5 Supplementary photos of the PhyTac device, including a larger one and a small one.

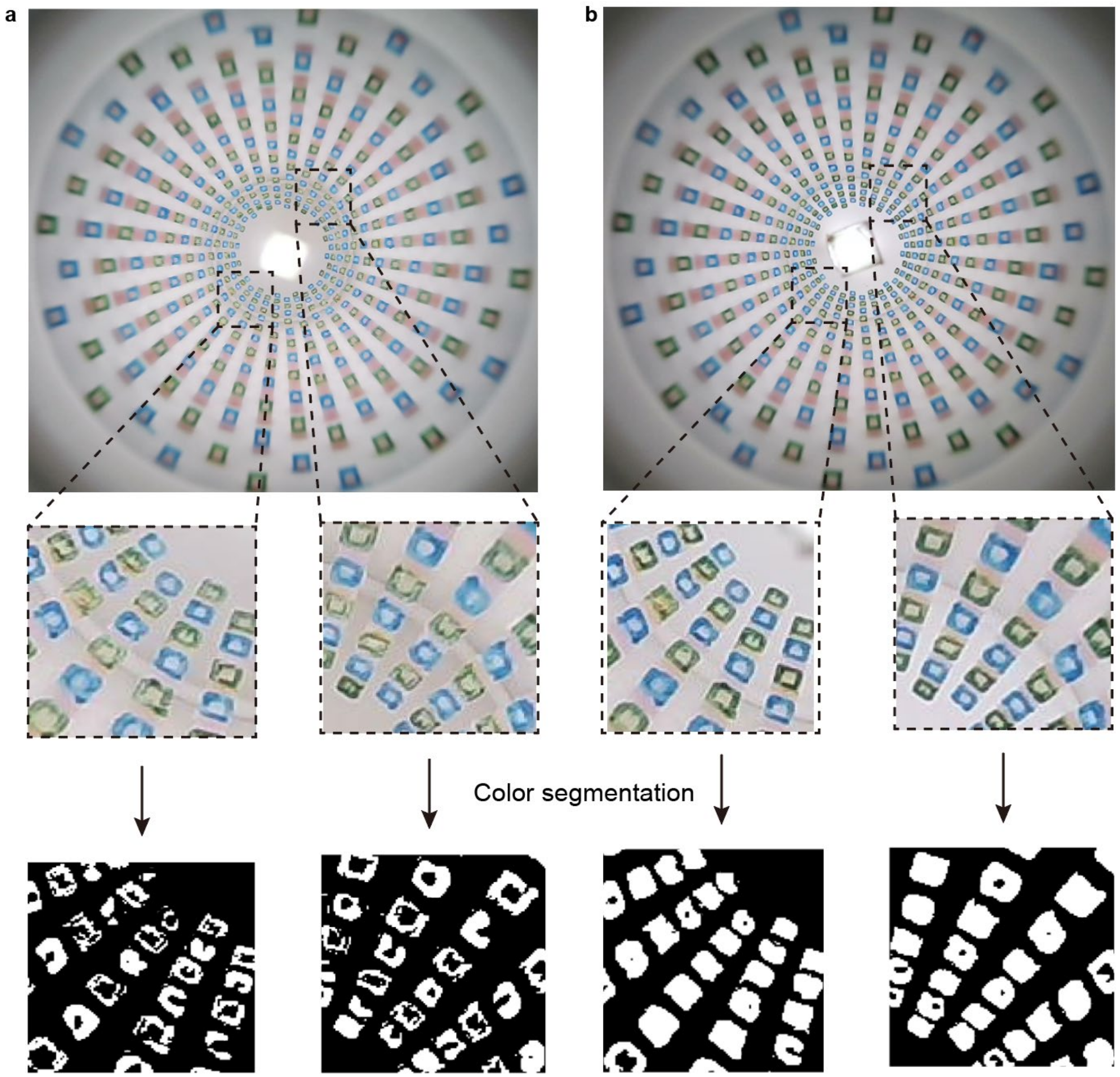

Figure S6 Comparison of different light sources. **a,** Linear light source without polarizing film, from which there is unwanted light refraction on the surface of markers and the segmentation results are not as well as expected. **b,** Linear light source with polarizing films, from which light refraction is eliminated and all markers can be segmented clearly in image processing.

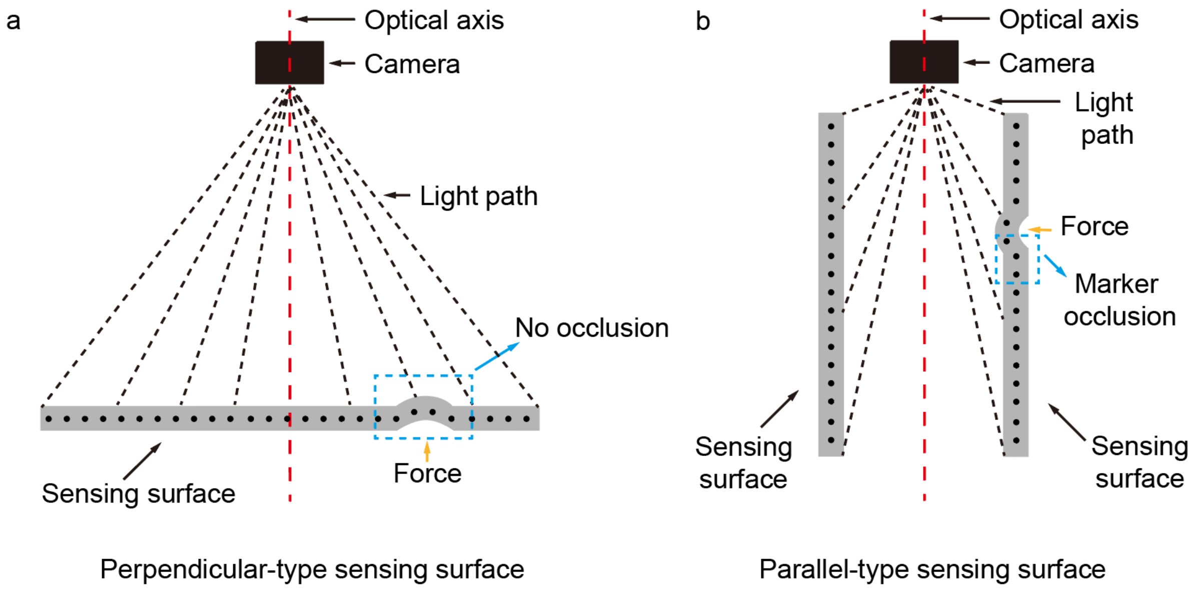

Figure S7 Comparison of two types of sensing surfaces. a, perpendicular-type sensing surfaces: there is almost no marker occlusion when deformation. **b,** parallel-type sensing surfaces: marker occlusions are common when deformation.

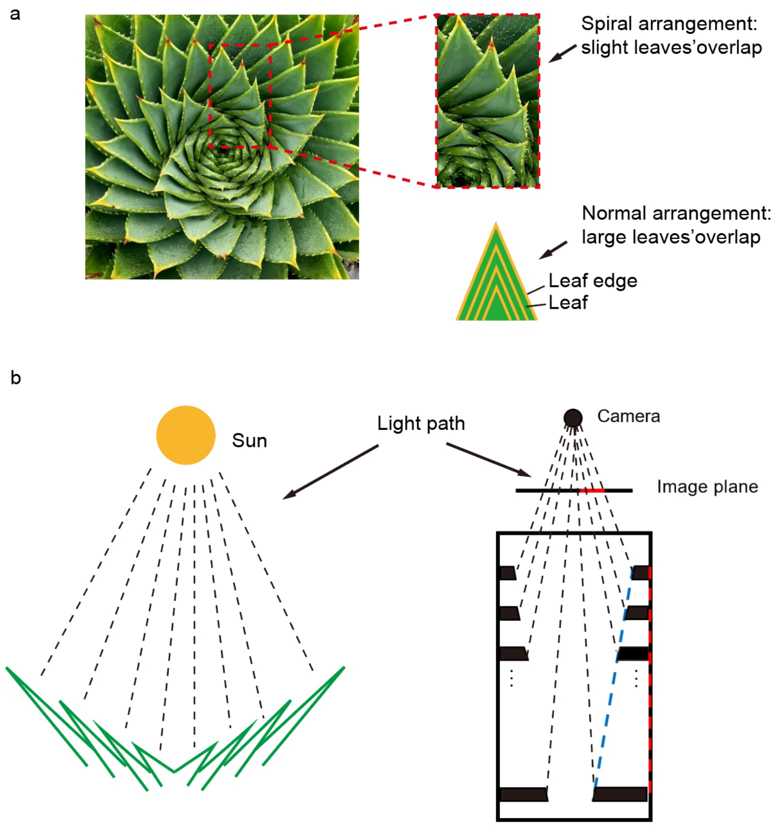

Figure S8 Spiral leaves can optimize the light path arrangement by avoiding leaf occlusion or self-shading, which is very instructive to the camera-based sensor design. a, Comparison of spiral arranged leaves and normal arranged leaves. b, Comparison of light paths between aloe polyphylla and PhyTac.

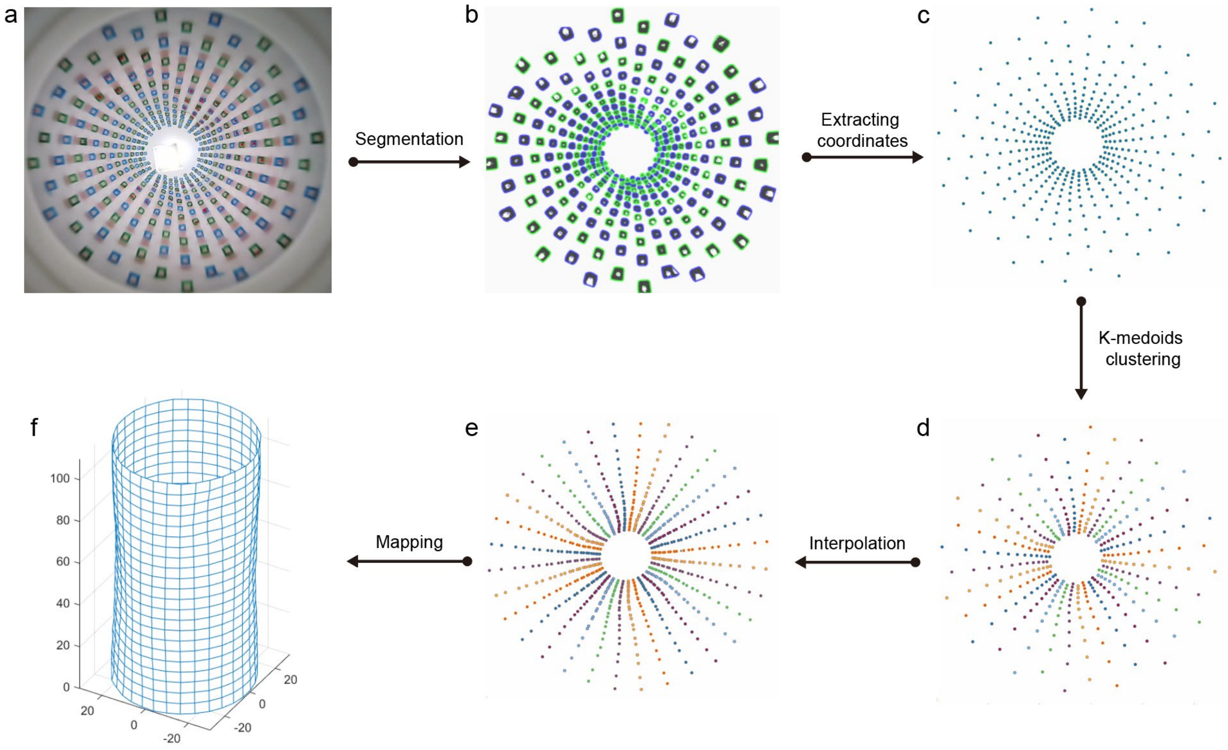

Figure S9 The procedures of 3D reconstruction. **a**, The dynamic image. **b**, Marker segmentation results. **c**, Extracted marker coordinates in image coordinate system. **d**, Grouped coordinates by column. **e**, Linear interpolation. **f**, Reconstructed outer shell in world coordinate system after coordinate mapping.

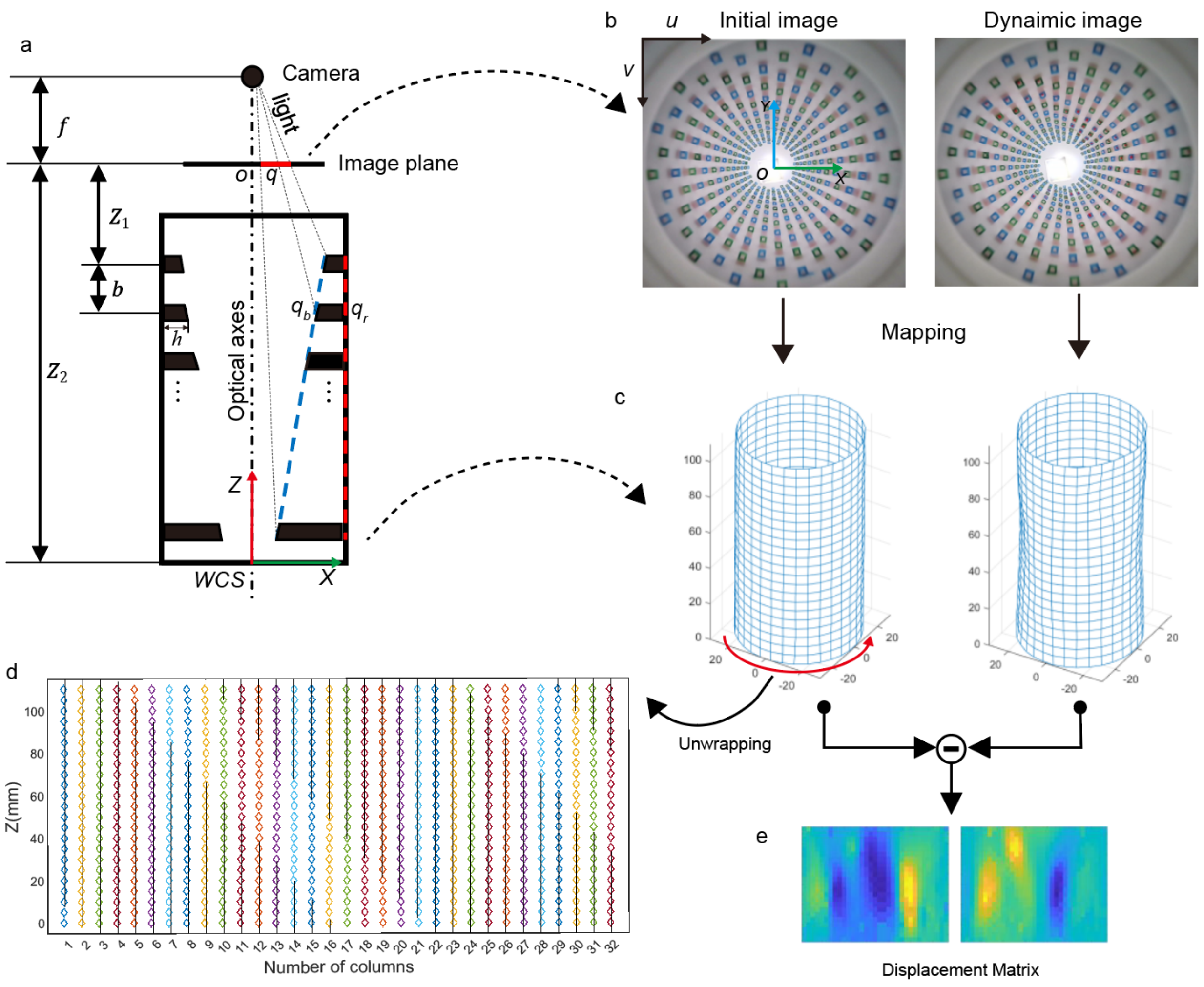

Figure S10 The Geometric and mapping relationship between image coordinate system and world coordinate system. **a**, Geometric layout of the camera and the outer shell of PhyTac. **b**, Captured initial image and dynamic image. **c**, Reconstructed 3D models of the initial state and dynamic state. **d**, Initial coordinates unwrapped in the circumferential direction. **e**, The calculated displacement matrix in X and Y directions.

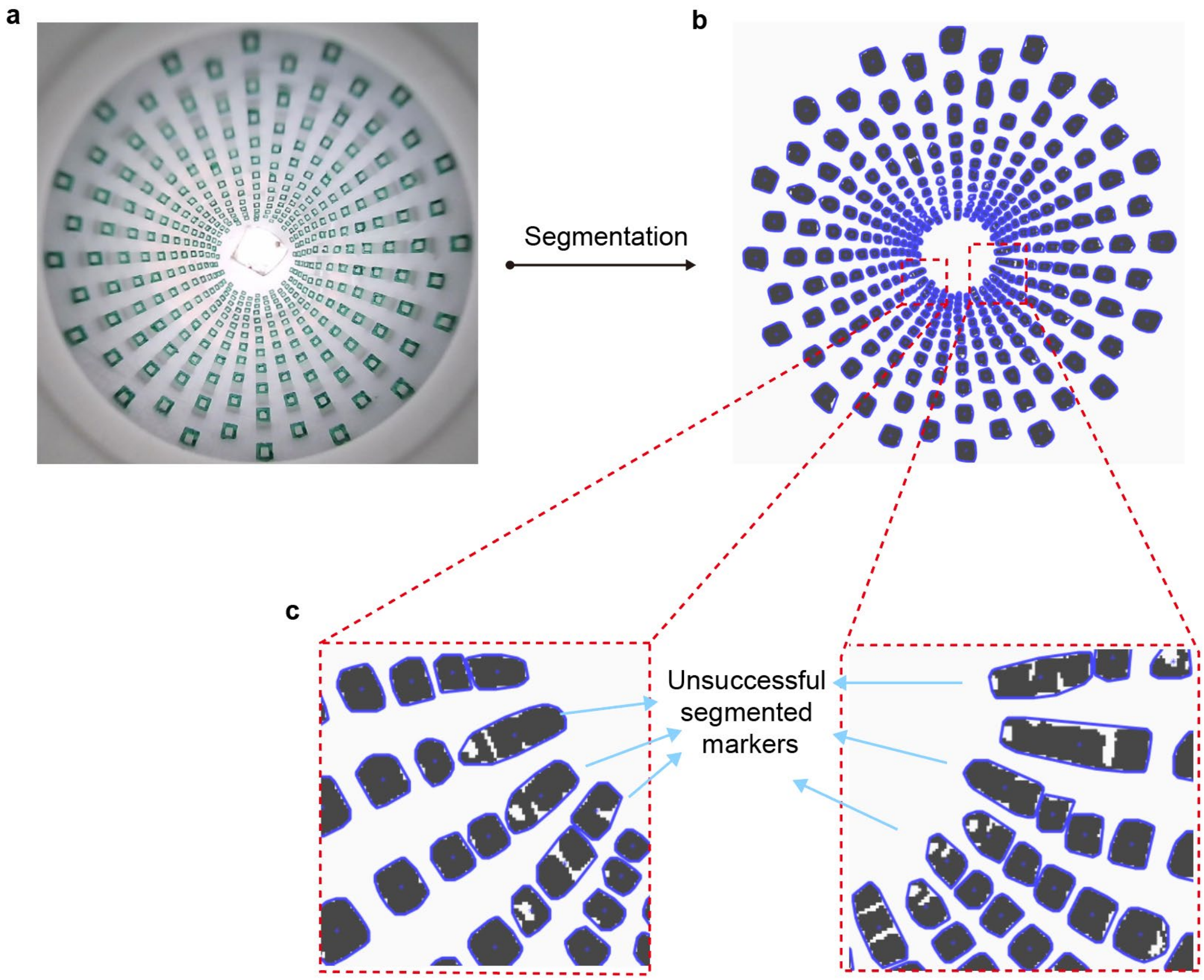

Figure S11 Processing results of traditional image. **a**, The image with traditional color distribution. **b**, The processing results of color segmentation. **c**, Partial enlarged details of unsuccessful segmented marker.

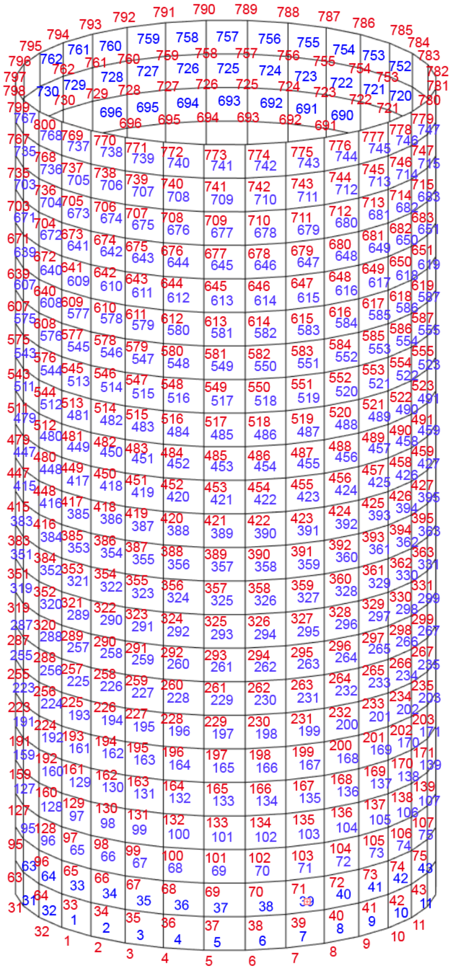

Figure S12 Finite element mesh of the outer shell of the PhyTac. The red number represents the number of nodes, and the blue number represents the number of elements.

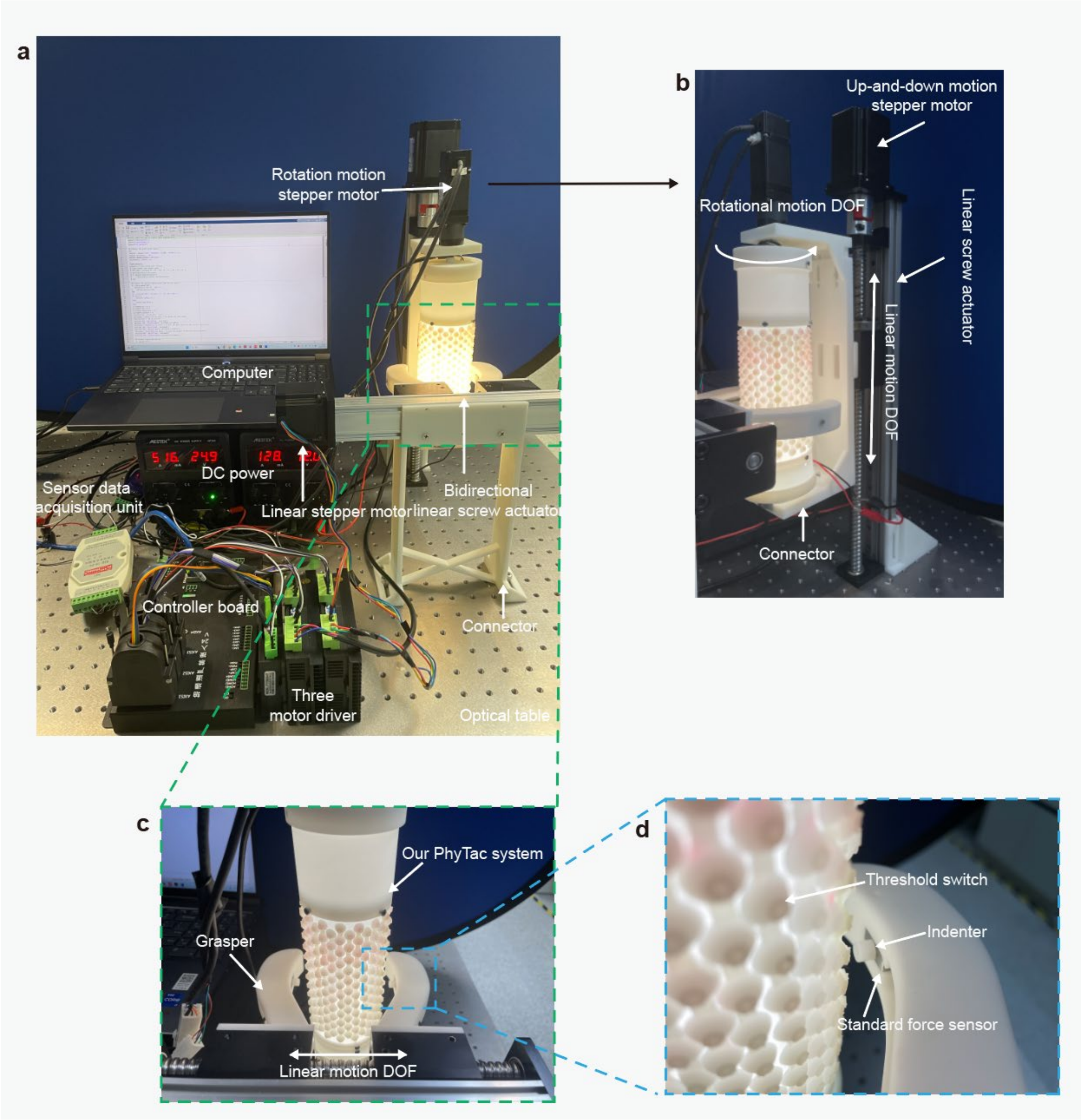

Figure S13 The configuration of 3-DOF automatic test platform. **a**, Overview layout of the test platform and its components. **b**, Details of the rotational motion DOF and the linear motion DOF. **c**, Details of the second linear motion DOF and the grasper. **d**, Details of the single-point contact assembly of the standard force sensor and indenter.

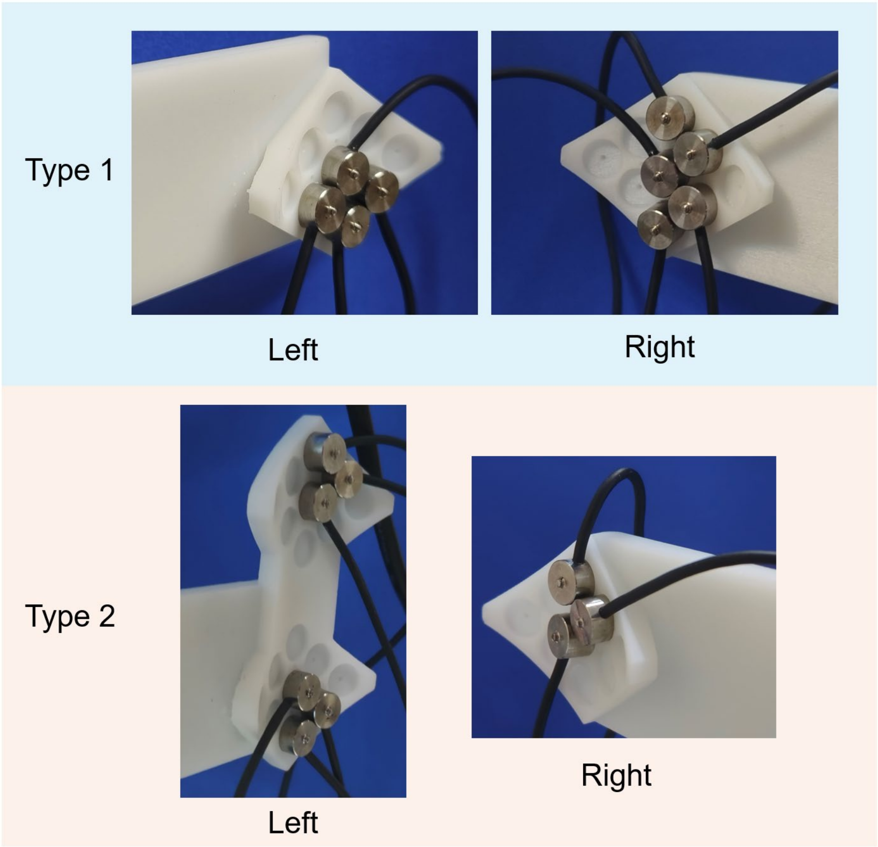

Figure S14 Two types of multiple point contact grippers and sensor distribution

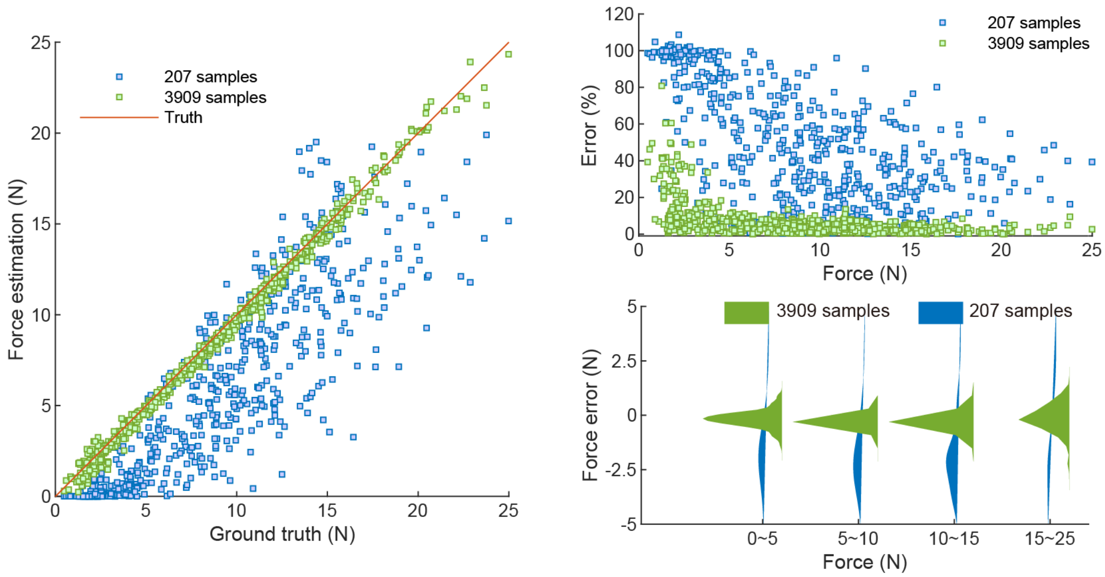

Figure S15 Accuracy comparison of CNN models with small and large datasets

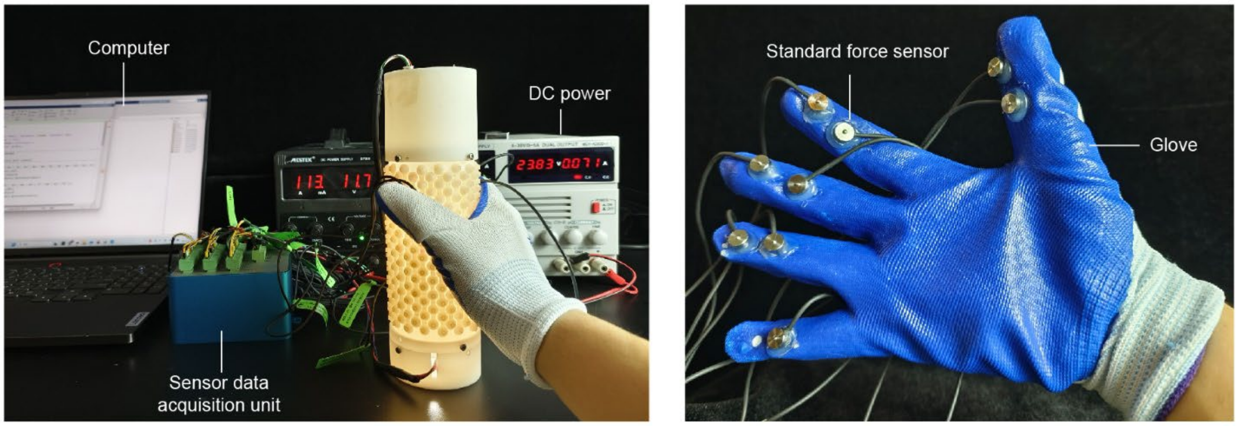

Figure S16 Validation platform to collect data of real hand grip

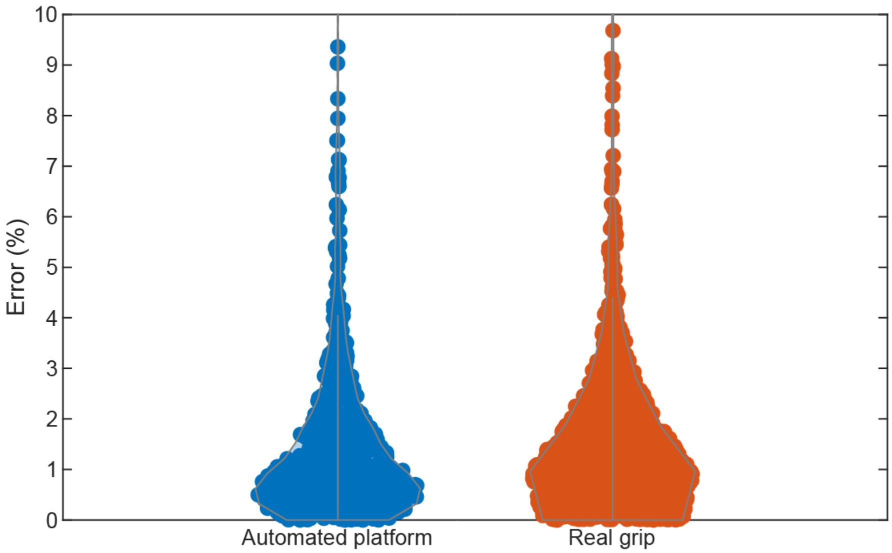

Figure S17 Overall accuracy comparison between automated platform and real hand grip

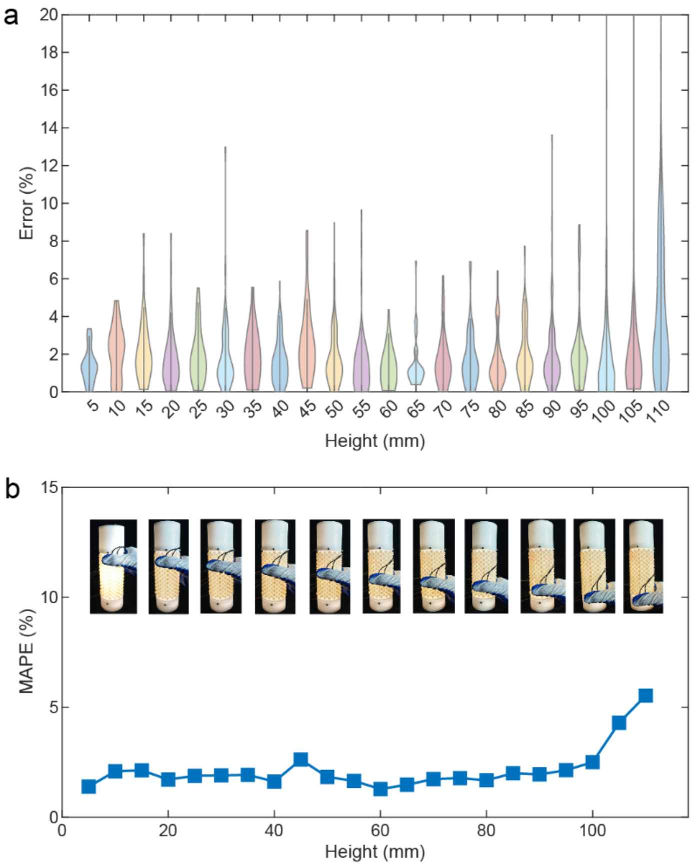

Figure S18 Device performance in different heights. a, The relative force error distribution in different heights. **b,** The quantitative evaluation among different rotational angles

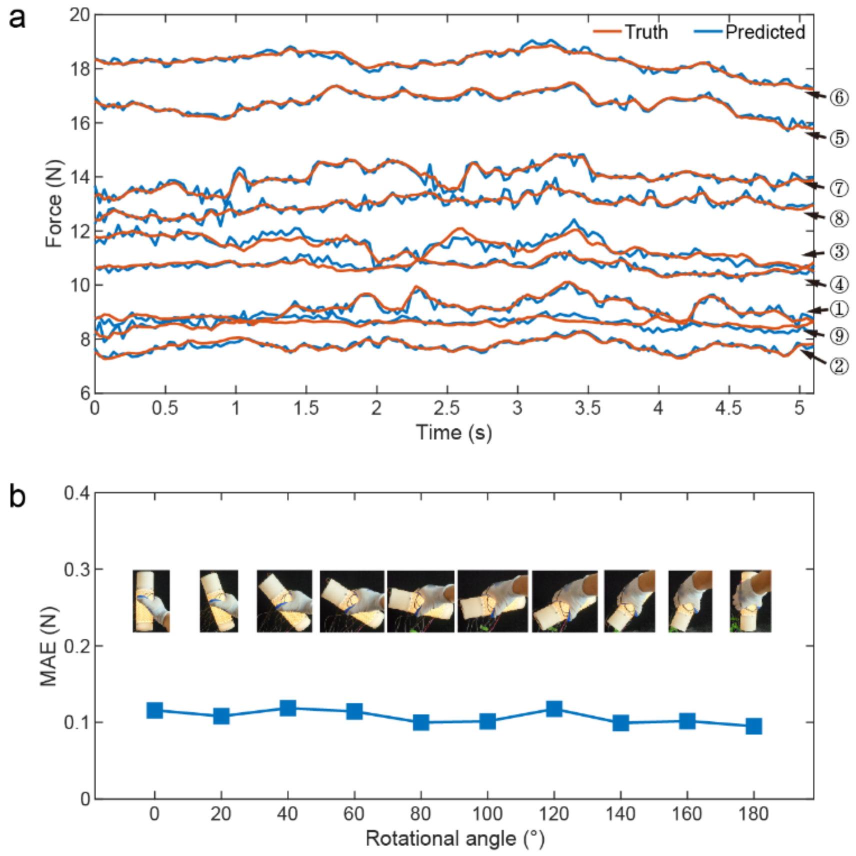

Figure S19 Device performance in different orientations. a, Force prediction when changing z-axis orientation. **b,** Error comparison among different rotational angles.

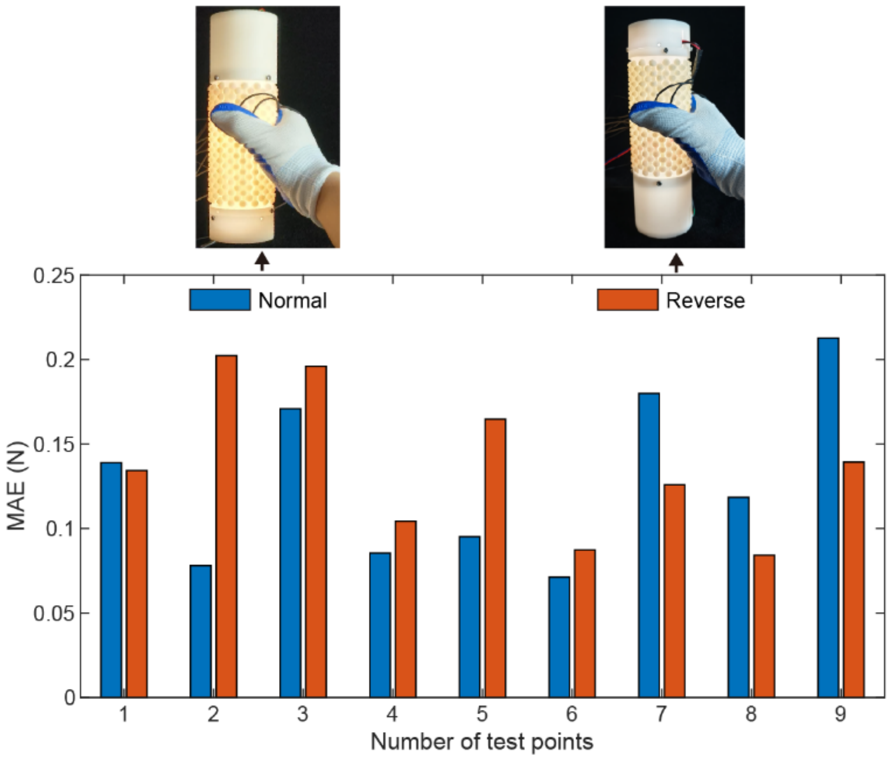

Figure S20 Performance comparison between reversed and normal grip direction.

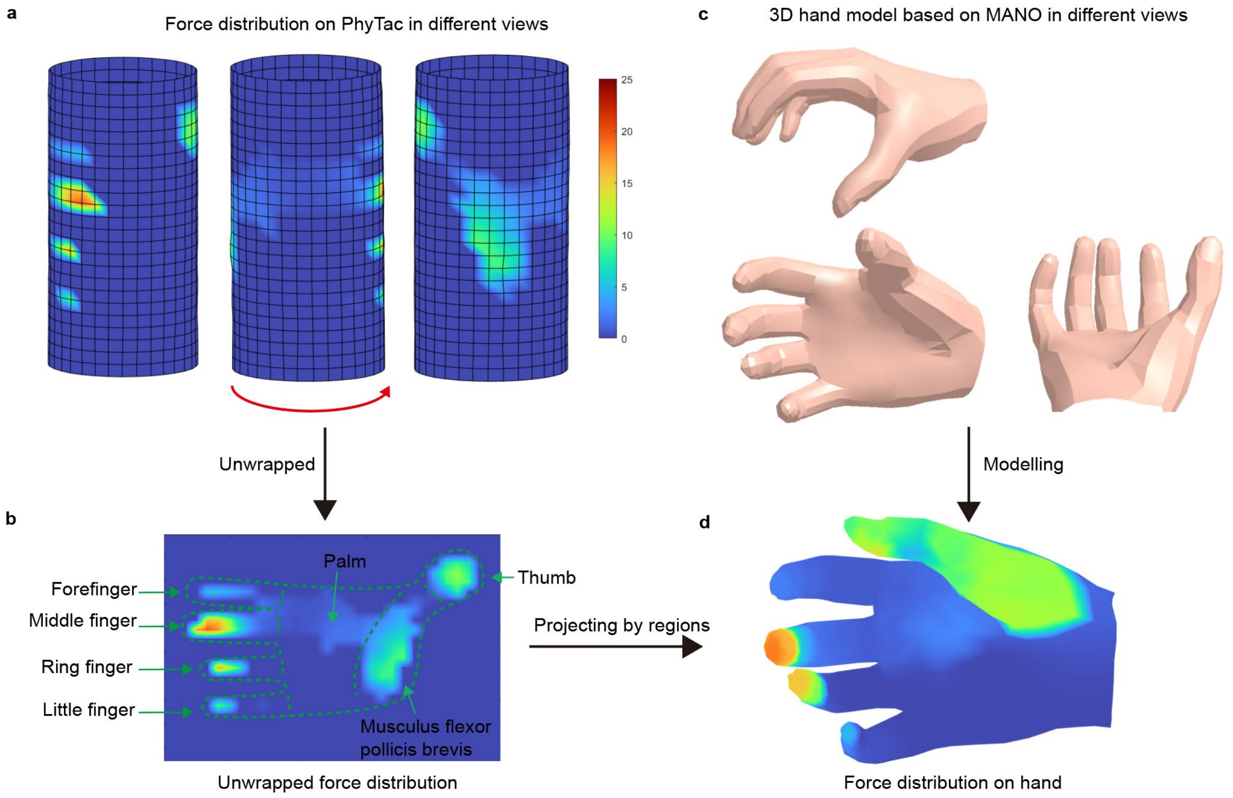

Figure S21 The procedures of projecting force distribution from PhyTac to the hand. a, The original force distribution on PhyTac in three views**. b,** Unwrapped force distribution, which can be divided into seven different regions, including forefinger, middle finger, ring finger, little finger, thumb, musculus flexor pollicis brevis and palm. **c,** The 3D hand model based on MANO *(49)* in three views. **d**, hand based force distribution.

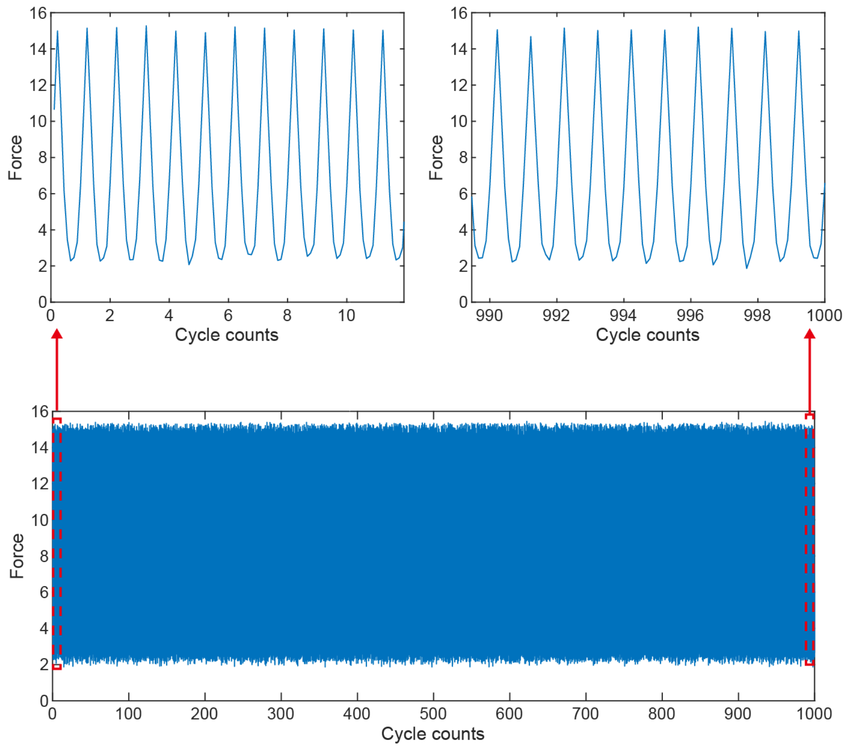

Figure S22 Working stability tested over 1,000 cycles

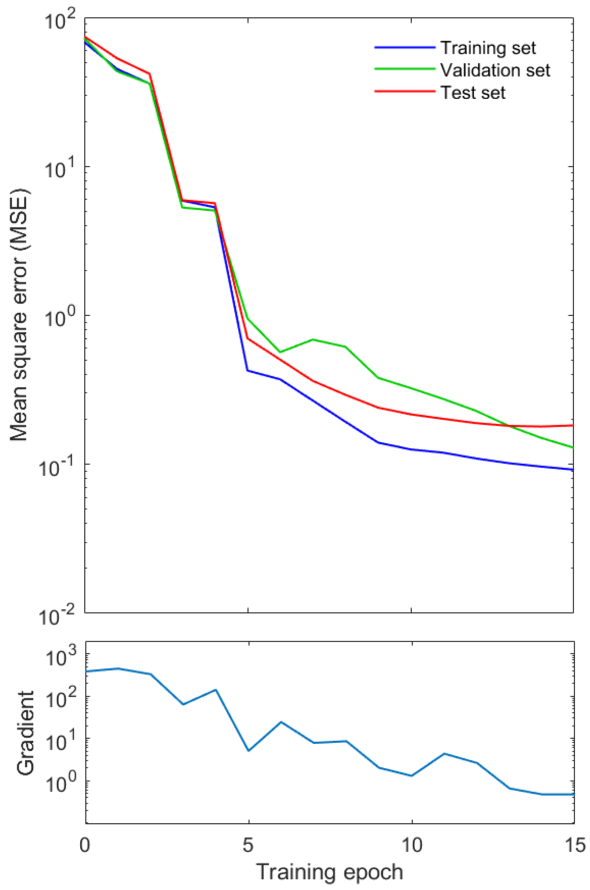

Figure S23 Training process of the physical model enhanced neural network.

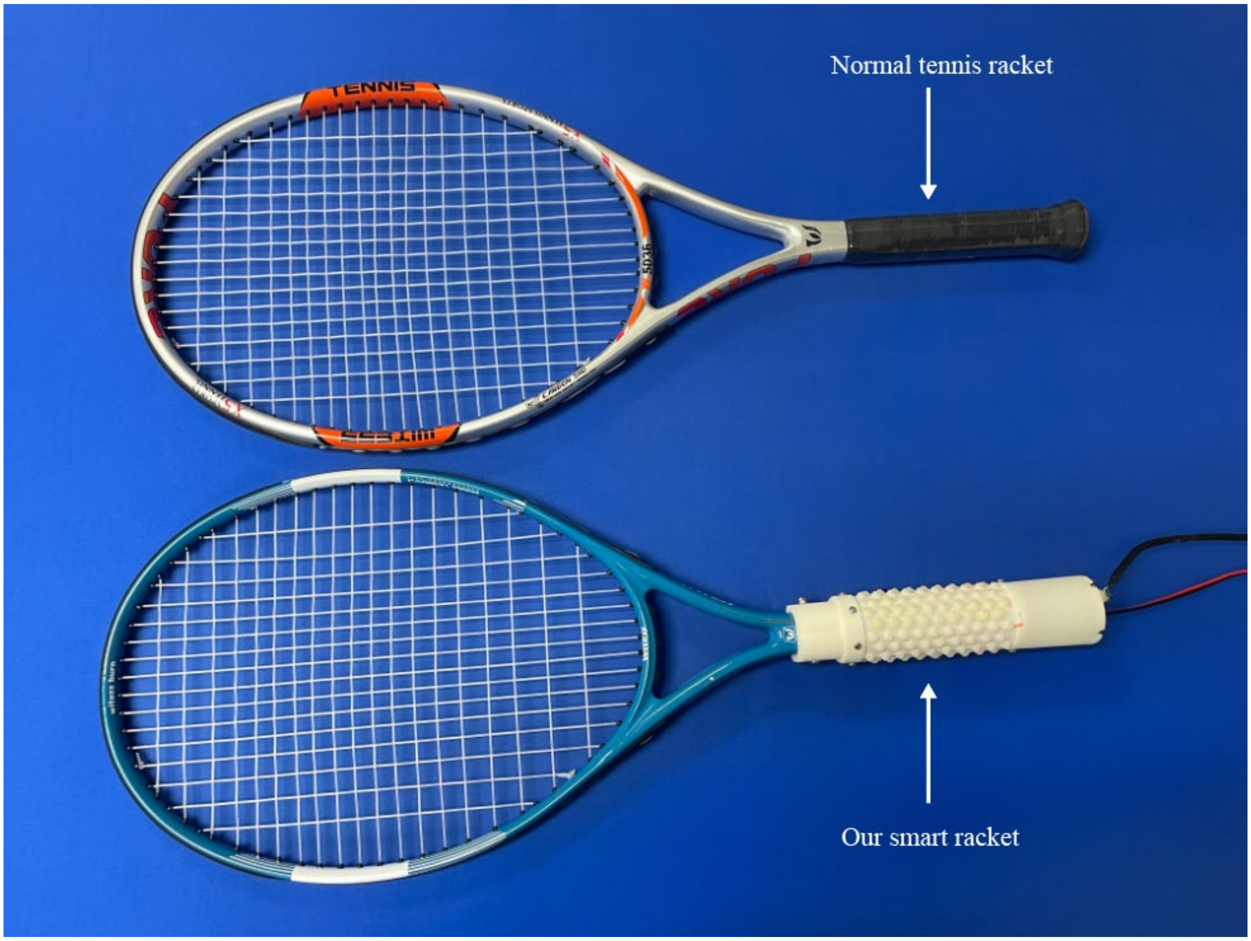

Figure S24 Comparison of the smart racket (integration with PhyTac) and the normal tennis racket.

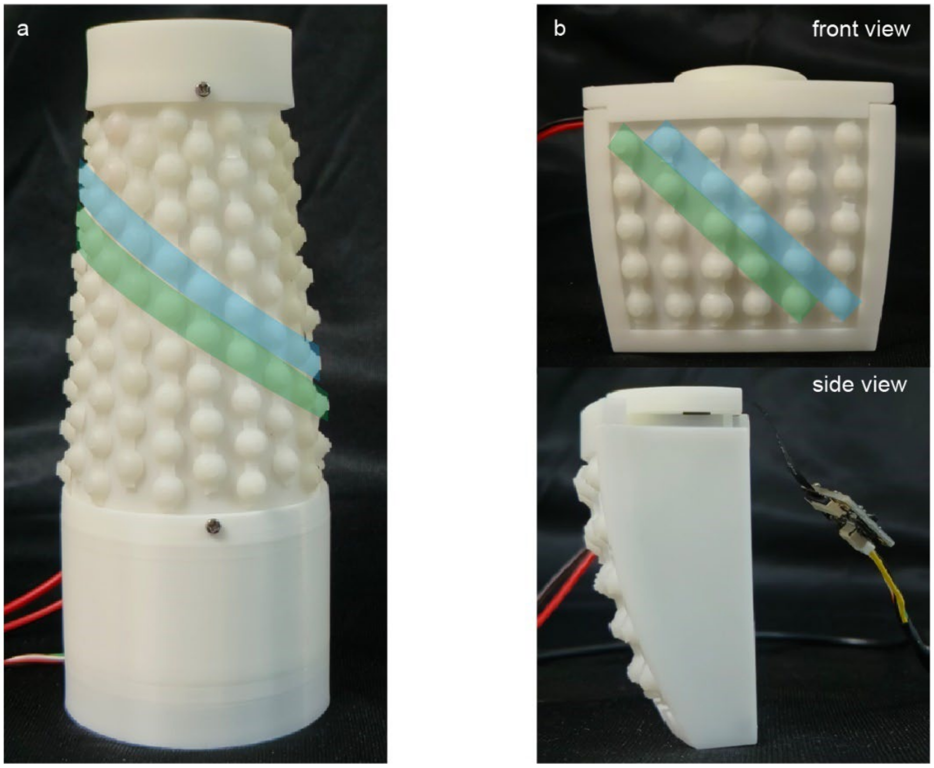

Figure S25 Devices with different geometries. a, device with cone-like surface. **b,** device with curved surface.

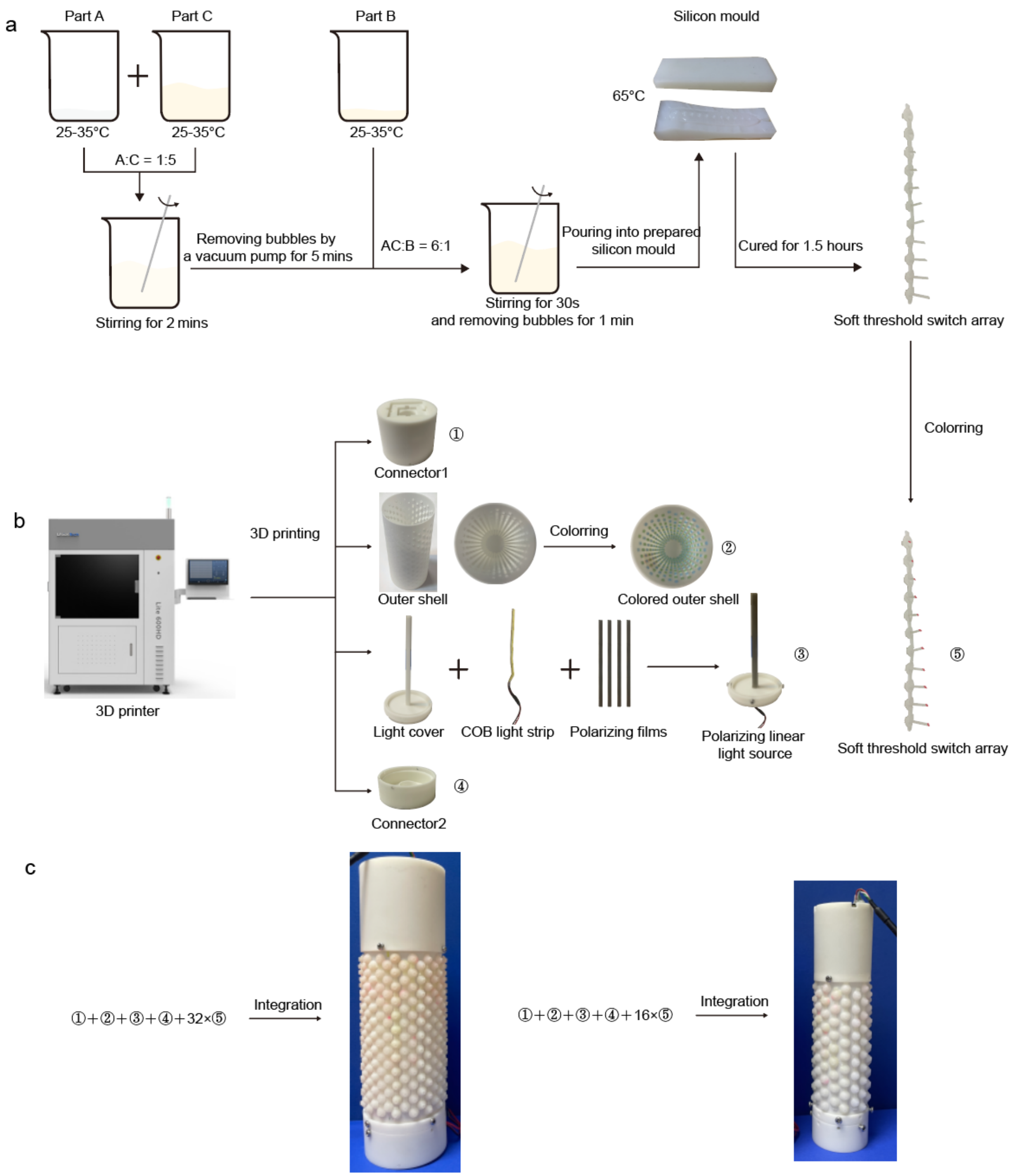

Figure S26 The procedures of fabrication and assembly of the PhyTac. a, Fabrication process of the soft threshold switch array. **b,** Fabrication process of the outer shell, light source and connectors **c**, Assembly results.

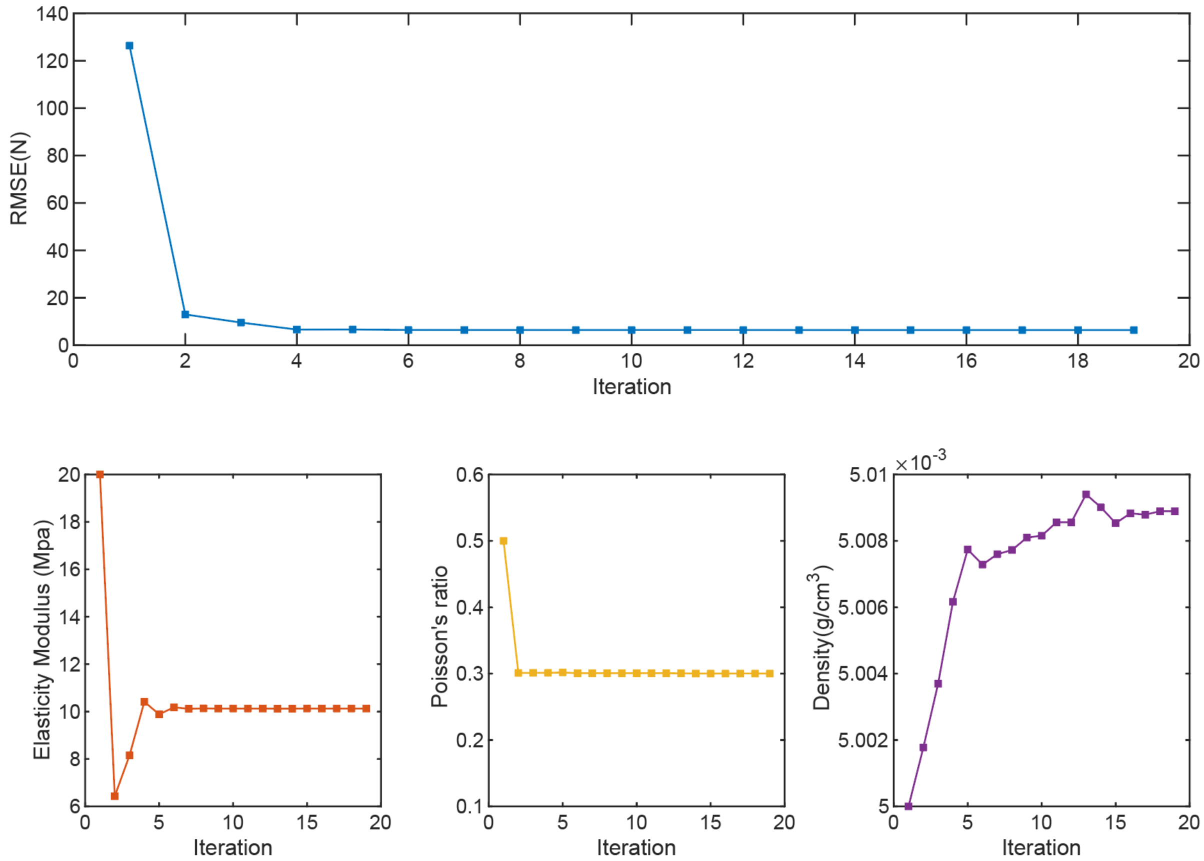

Figure S27 Calibration optimization of Finite element model for force estimation. The RMSE is the objective function, and the elasticity modulus, the Poisson's ratio and the density are the parameters to be optimized. These parameters are identified simultaneously, and the optimization is based on a nonlinear optimizer (fmincon in MATLAB).

Table S1 Comparison with commonly used dynamometers evaluating force generated hand

**Sensing type**	**Principle**	**Spatial information**	**Temporal information**	**Advantages**	**Issues**
Hydraulic *(50)*	Force changes the hydraulic pressure in a sealed hydraulic system	Only maximum grip strength	Only one dimension	Portable, economical, accurate	No force distribution
Pneumatic *(50)*	Force compresses the air pressure in an air-filled bag or bulb	Only maximum grip strength	Only one dimension	Gentler on weak or painful joints of hand	No force distribution
Mechanical *(51)*	The amount of tension produced in a spring	Only maximum grip strength	Only one dimension	Portable, economical	No force distribution, inaccurate
Electronic *(16)*	Resistive/ capacitive/ magnetic piezoelectric/ triboelectric	Force distribution	Multiple dimensions	Force distribution	Expensive, mass wirings, sensitive to environment
PhyTac (Our work)	Force changes light path/ reflection/ refraction, and further changes the captured image by camera	Force distribution	Multiple dimensions	Accurate, economical, force distribution, robust, gentler on weak joints	-

Table S2. Comparison with other vision-based methods

Method	Sensor	Morpho-logy	Area (mm^2)	Force map	Dataset	Maximum range (N)	Force error (%)	Type-1 Marker density	Type-2 Marker density
Markers + Data-driven	Gelsight *(22)*	2D	250	No	4 GB*	25	>7.2*	81/cm^2	-
	C.S. et al. *(33)*	2D	900	No	2.4 GB*	16	3.4*	>200/cm^2	-
	DIGIT *(52)*	2D	304	No	56 MB*	-	-	>50/cm^2	-
	Tactip *(24,53)*	3D	16,221	No	1.2 GB*	-	-	>2.7/cm^2	1.1/cm^2
	SimTacLS *(26)*	3D	36,534	No	2 GB*	-	-	-	0.7/cm^2
Markers + FEM	GelSlim *(31)*	2D	1,200	Yes	-	4	15	>20/cm^2	-
	TacLINK *(25)*	3D	57,776	Yes	-	1.5	>20*	-	0.42/cm^2
Physical model	GelTip *(54)*	3D	2,513	No	-	-	-	-	-
Data-driven	OmniTact *(55)*	3D	3,110	No	N.A.	-	-	-	-
	Insight *(27)*	3D	4,800	Yes	11.2 GB	2	>4	-	-
Markers + Physical model enhanced neural network (This work)	PhyTac	3D	22,608	Yes	**45 KB**	**25**	**2.3**	-	**1.63/cm^2**

Note : * represents that this value is calculated by the published data in this paper; Type-1 refers to perpendicular-type sensing surfaces; Type-2 refers to parallel-type sensing surfaces.”

Table S3 Comparison of existing teleoperation methods

Literature	Device	Input to robotic hand	Weight	Cost	Avg time of pick-and-place
(56)	Joysticks	Positions	N/A	N/A	~450 s
(57)	Curvature sensors + Glove + IMUs	Finger curvatures	213g	<$150	~34.5 s
(58)	Exoskeleton + angle sensors	Finger angles	350g	N/A	~12.2 s
(59)	Cyberglove + Motion Capture	Hand motion	>540g	>$13,000	Not Reported
(60)	P5 Virtual Reality Glove	Finger angles	~127g	>$250	Not Reported
Our work	PhyTac + IMUs	Distributed force	139g	<$100	~50 s

Movie descriptions

Movie S1
Digital channel enabled force localization and real-time marker segmentation when hand makes interaction with the PhyTac, from which we can see that force location can be easily identified by digital channel, and contours of all markers with different colors can be clearly segmented without marker fusion. Left: captured original image; Right: processed image.

Movie S2
Demonstration of PhyTac in application of hand evaluation, for providing grip strength distribution information when the user orderly uses finger to apply force. Left: two views with different viewing angle; Right top: the captured original image; Right bottom: the real-time force distribution on hand.

Movie S3
Demonstration of PhyTac in application of smart sport equipment, for providing force distribution when user hit a tennis ball using forehand and backhand. The applied force of forehand is distributed in the forefinger, middle finger and ring finger and their surrounding part of palm when hitting. By contrast, the applied force of backhand is distributed in the thumb, ring finger and little finger and their surrounding part of palm when hitting.

Movie S4
Demonstration of PhyTac in application of VR interactions, where the user grasps, holds and crushes an egg and a vase, and deforms a soft ball by projecting force distribution in real world to virtual world.

Movie S5
Demonstration of PhyTac in application of teleoperation and human-robot interactions, where user uses PhyTac (integrating with an IMU) to control the movement/rotation of the robot arm and the precise grasp of the robotic hand.

REFERENCES AND NOTES

1. Q. Li, O. Kroemer, Z. Su, F. F. Veiga, M. Kaboli, H. J. Ritter, A review of tactile information: Perception and action through touch. *IEEE Trans. Robot.* **36**, 1619–1634 (2020).

2. I. You, D. G. Mackanic, N. Matsuhisa, J. Kang, J. Kwon, L. Beker, J. Mun, W. Suh, T. Y. Kim, J. B.-H. Tok, Z. Bao, U. Jeong, Artificial multimodal receptors based on ion relaxation dynamics. *Science* **370**, 961–965 (2020).

3. L. Chen, S. Karilanova, S. Chaki, C. Wen, L. Wang, B. Winblad, S.-L. Zhang, A. Özçelikkale, Z.-B. Zhang, Spike timing–based coding in neuromimetic tactile system enables dynamic object classification. *Science* **384**, 660–665 (2024).

4. Z. Liu, X. Hu, R. Bo, Y. Yang, X. Cheng, W. Pang, Q. Liu, Y. Wang, S. Wang, S. Xu, Z. Shen, Y. Zhang, A three-dimensionally architected electronic skin mimicking human mechanosensation. *Science* **384**, 987–994 (2024).

5. C. Xu, Y. Wang, J. Zhang, J. Wan, Z. Xiang, Z. Nie, J. Xu, X. Lin, P. Zhao, Y. Wang, S. Zhang, J. Zhang, C. Liu, N. Xue, W. Zhao, M. Han, Three-dimensional micro strain gauges as flexible, modular tactile sensors for versatile integration with micro- and macroelectronics. *Sci. Adv.* **10**, eadp6094 (2024).

6. R. L. Klatzky, S. Lederman, “Intelligent exploration by the human hand” in *Dextrous Robot Hands*, S. T. Venkataraman, T. Iberall, Eds. (Springer, 1990; https://doi.org/10.1007/978-1-4613-8974-3_4), pp. 66–81.

7. R. J. K. Jacob, Human-computer interaction: Input devices. *ACM Comput. Surv.* **28**, 177–179 (1996).

8. R.-D. Vatavu, “Gesture-based interaction,” in *Handbook of Human Computer Interaction*, J. Vanderdonckt, P. Palanque, M. Winckler, Eds. (Springer International Publishing, 2020; https://doi.org/10.1007/978-3-319-27648-9_20-1), pp. 1–47.

9. R. W. McGorry, P. G. Dempsey, J. S. Casey, The effect of force distribution and magnitude at the hand-tool interface on the accuracy of grip force estimates. *J. Occup. Rehabil.* **14**, 255–266 (2004).

10. L. Burnie, N. Chockalingam, A. Holder, T. Claypole, L. Kilduff, N. Bezodis, Commercially available pressure sensors for sport and health applications: A comparative review. *Foot* **56**, 102046 (2023).

11. Y. Luo, Y. Li, P. Sharma, W. Shou, K. Wu, M. Foshey, B. Li, T. Palacios, A. Torralba, W. Matusik, Learning human–environment interactions using conformal tactile textiles. *Nat. Electron.* **4**, 193–201 (2021).

12. Y. Shi, F. Wang, J. Tian, S. Li, E. Fu, J. Nie, R. Lei, Y. Ding, X. Chen, Z. L. Wang, Self-powered electro-tactile system for virtual tactile experiences. *Sci. Adv.* **7**, eabe2943 (2021).

13. J. A. Barreiros, A. Xu, S. Pugach, N. Iyengar, G. Troxell, A. Cornwell, S. Hong, B. Selman, R. F. Shepherd, Haptic perception using optoelectronic robotic flesh for embodied artificially intelligent agents. *Sci. Robot.* **7**, eabi6745 (2022).

14. Y. Yan, Z. Hu, Z. Yang, W. Yuan, C. Song, J. Pan, Y. Shen, Soft magnetic skin for super-resolution tactile sensing with force self-decoupling. *Sci. Robot.* **6**, eabc8801 (2021).

15. J. Shi, Y. Dai, Y. Cheng, S. Xie, G. Li, Y. Liu, J. Wang, R. Zhang, N. Bai, M. Cai, Y. Zhang, Y. Zhan, Z. Zhang, C. Yu, C. F. Guo, Embedment of sensing elements for robust, highly sensitive, and cross-talk–free iontronic skins for robotics applications. *Sci. Adv.* **9**, eadf8831 (2023).

16. H. C. Roberts, H. J. Denison, H. J. Martin, H. P. Patel, H. Syddall, C. Cooper, A. A. Sayer, A review of the measurement of grip strength in clinical and epidemiological studies: Towards a standardised approach. *Age Ageing* **40**, 423–429 (2011).

17. S. Sundaram, P. Kellnhofer, Y. Li, J.-Y. Zhu, A. Torralba, W. Matusik, Learning the signatures of the human grasp using a scalable tactile glove. *Nature* **569**, 698–702 (2019).

18. X.-M. Wang, L.-Q Tao, M. Yuan, Z.-P Wang, J. Yu, D. Xie, F. Luo, X. Chen, C. Wong, Sea urchin-like microstructure pressure sensors with an ultra-broad range and high sensitivity. *Nat. Commun.* **12**, 1776 (2021).

19. Y. Tang, T. Zhang, H. Ren, W. Zhang, G. Li, D. Guo, L. Yang, R. Tan, Y. Shen, Highly sensitive spherical cap structure-based iontronic pressure sensors by a mold-free fabrication approach. *Smart Mater. Struct.* **31**, 095030 (2022).

20. J. Li, H. Jia, J. Zhou, X. Huang, L. Xu, S. Jia, Z. Gao, K. Yao, D. Li, B. Zhang, Y. Liu, Y. Huang, Y. Hu, G. Zhao, Z. Xu, J. Li, C. K. Yiu, Y. Gao, M. Wu, Y. Jiao, Q. Zhang, X. Tai, R. H. Chan, Y. Zhang, X. Ma, X. Yu, Thin, soft, wearable system for continuous wireless monitoring of artery blood pressure. *Nat. Commun.* **14**, 5009 (2023).

21. J. Li, Y. Liu, M. Wu, K. Yao, Z. Gao, Y. Gao, X. Huang, T. H. Wong, J. Zhou, D. Li, H. Li, J. Li, Y. Huang, R. Shi, J. Yu, X. Yu, Thin, soft, 3D printing enabled crosstalk minimized triboelectric nanogenerator arrays for tactile sensing. *Fundam. Res.* **3**, 111–117 (2023).

22. W. Yuan, S. Dong, E. H. Adelson, GelSight: High-resolution robot tactile sensors for estimating geometry and force. *Sensors* **17**, 2762 (2017).

23. S. Wang, Y. She, B. Romero, E. Adelson, "GelSight wedge: Measuring high-resolution 3D contact geometry with a compact robot finger" in *2021 IEEE International Conference on Robotics and Automation (ICRA)* (IEEE, 2021; https://ieeexplore.ieee.org/document/9560783), pp. 6468–6475.

24. B. Ward-Cherrier, N. Pestell, L. Cramphorn, B. Winstone, M. E. Giannaccini, J. Rossiter, N. F. Lepora, The TacTip family: Soft optical tactile sensors with 3D-printed biomimetic morphologies. *Soft Robot.* **5**, 216–227 (2018).

25. L. Van Duong, V. A. Ho, Large-scale vision-based tactile sensing for robot links: Design, modeling, and evaluation. *IEEE Trans. Robot.* **37**, 390–403 (2021).

26. Q. K. Luu, N. H. Nguyen, V. A. Ho, Simulation, learning, and application of vision-based tactile sensing at large scale. *IEEE Trans. Robot.* **39**, 2003–2019 (2023).

27. H. Sun, K. J. Kuchenbecker, G. Martius, A soft thumb-sized vision-based sensor with accurate all-round force perception. *Nat. Mach. Intell.* **4**, 135–145 (2022).

28. W.-G. Kim, D.-W. Kim, I.-W. Tcho, J.-K. Kim, M.-S. Kim, Y.-K. Choi, Triboelectric nanogenerator: Structure, mechanism, and applications. *ACS Nano* **15**, 258–287 (2021).

29. Y. Chang, L. Wang, R. Li, Z. Zhang, Q. Wang, J. Yang, C. F. Guo, T. Pan, First decade of interfacial iontronic sensing: From droplet sensors to artificial skins. *Adv. Mater.* **33**, 2003464 (2021).

30. H. Bai, S. Li, J. Barreiros, Y. Tu, C. R. Pollock, R. F. Shepherd, Stretchable distributed fiber-optic sensors. *Science* **370**, 848–852 (2020).

31. D. Ma, E. Donlon, S. Dong, A. Rodriguez, Dense Tactile Force Estimation using GelSlim and inverse FEM, in *2019 International Conference on Robotics and Automation (ICRA)* (2019), pp. 5418–5424.

32. C. Sferrazza, R. D'Andrea, Design, motivation and evaluation of a full-resolution optical tactile sensor. *Sensors* **19**, 928 (2019).

33. C. Sferrazza, R. D'Andrea, Sim-to-real for high-resolution optical tactile sensing: From images to three-dimensional contact force distributions. *Soft Robotics* **9**, 926–937 (2022).

34. A. Handler, D. D. Ginty, The mechanosensory neurons of touch and their mechanisms of activation. *Nat. Rev. Neurosci.* **22**, 521–537 (2021).

35. N. L. Neubarth, A. J. Emanuel, Y. Liu, M. W. Springel, A. Handler, Q. Zhang, B. P. Lehnert, C. Guo, L. L. Orefice, A. Abdelaziz, M. M. DeLisle, M. Iskols, J. Rhyins, S. J. Kim, S. J. Cattel, W. Regehr, C. D. Harvey, J. Drugowitsch, D. D. Ginty, Meissner corpuscles and their spatially intermingled afferents underlie gentle touch perception. *Science* **368**, eabb2751 (2020).

36. D. Reinhardt, E. M. Gola, Law and order in plants—The origin and functional relevance of phyllotaxis. *Trends Plant Sci.* **27**, 1017–1032 (2022).

37. S. Strauss, J. Lempe, P. Prusinkiewicz, M. Tsiantis, R. S. Smith, Phyllotaxis: Is the golden angle optimal for light capture? *New Phytol.* **225**, 499–510 (2020).

38. R. W. Pearcy, W. Yang, The functional morphology of light capture and carbon gain in the Redwood forest understorey plant Adenocaulon bicolor Hook. *Funct. Ecol.* **12**, 543–552 (1998).

39. S. King, F. Beck, U. Lüttge, On the mystery of the golden angle in phyllotaxis. *Plant Cell Environ.* **27**, 658–695 (2004)

40. N. J. Seo, T. J. Armstrong, Investigation of grip force, normal force, contact area, hand size, and handle size for cylindrical handles. *Hum. Factors* **50**, 734–744 (2008).

41. H. Wang, W. Wang, J. J. Kim, C. Wang, Y. Wang, B. Wang, S. Lee, T. Yokota, T. Someya, An optical-based multipoint 3-axis pressure sensor with a flexible thin-film form. *Sci. Adv.* **9**, eadi2445 (2023).

42. S. Herbort, A. Grumpe, C. Wöhler, Reconstruction of non-Lambertian surfaces by fusion of Shape from Shading and active range scanning, in *2011 18th IEEE International Conference on Image Processing* (2011), pp. 17–20.

43. E. Oñate, "Thick/thin plates. Reissner-Mindlin theory" in *Structural Analysis with the Finite Element Method Linear Statics: Volume 2. Beams, Plates and Shells*, E. Oñate, Ed. (Springer Netherlands, 2013; https://doi.org/10.1007/978-1-4020-8743-1_6) *Lecture Notes on Numerical Methods in Engineering and Sciences*, pp. 291–381.

44. G. E. Karniadakis, I. G. Kevrekidis, L. Lu, P. Perdikaris, S. Wang, L. Yang, Physics-informed machine learning. *Nat. Rev. Phys.* **3**, 422–440 (2021).

45. J. E. Mottershead, M. I. Friswell, Model updating in structural dynamics: A survey. *J. Sound Vib.* **167**, 347–375 (1993).

46. F. Salaffi, M. Carotti, S. Farah, L. Ceccarelli, M. Di Carlo, Handgrip strength features in rheumatoid arthritis patients assessed using an innovative cylindrical-shaped device: Relationships with demographic, anthropometric and clinical variables. *J. Med. Syst.* **45**, 100 (2021).

47. K. Zhang, K. Zhang, L. Wang, The evaluation of sports performance in tennis based on flexible piezoresistive pressure sensing technology. *IEEE Sens. J.* **24**, 28111–28118 (2024).

48. D. Chadefaux, G. Rao, J.-L. Le Carrou, E. Berton, L. Vigouroux, The effects of player grip on the dynamic behaviour of a tennis racket. *J. Sports Sci.* **35**, 1155–1164 (2017).

49. J. Romero, D. Tzionas, M. J. Black, Embodied hands: Modeling and capturing hands and bodies together. *ACM Trans. Graph.* **36**, 245 (2017).

50. J. Desrosiers, R. Hébert, G. Bravo, E. Dutil, Comparison of the Jamar dynamometer and the Martin vigorimeter for grip strength measurements in a healthy elderly population. *J. Rehabil. Med.* **27**, 137–143 (1995).

51. J. A. Balogun, S. A. Adenlola, A. A. Akinloye, Grip strength normative data for the harpenden dynamometer. *J. Orthop. Sports Phys. Ther.* **14**, 155–160 (1991).

52. M. Lambeta, P.-W. Chou, S. Tian, B. Yang, B. Maloon, V. R. Most, D. Stroud, R. Santos, A. Byagowi, G. Kammerer, D. Jayaraman, R. Calandra, DIGIT: A novel design for a low-cost compact high-resolution tactile sensor with application to in-hand manipulation. *IEEE Robot. Autom. Lett.* **5**, 3838–3845 (2020).

53. B. Winstone, C. Melhuish, T. Pipe, M. Callaway, S. Dogramadzi, Toward bio-inspired tactile sensing capsule endoscopy for detection of submucosal tumors. *IEEE Sens. J.* **17**, 848–857 (2017).

54. D. F. Gomes, Z. Lin, S. Luo, "GelTip: A finger-shaped optical tactile sensor for robotic manipulation" in *2020 IEEE/RSJ International Conference on Intelligent Robots and Systems (IROS)* (IEEE, 2020), pp. 9903–9909.

55. A. Padmanabha, F. Ebert, S. Tian, R. Calandra, C. Finn, S. Levine, "OmniTact: A multi-directional high-resolution touch sensor" in *2020 IEEE International Conference on Robotics and Automation (ICRA)* (IEEE, 2020), pp. 618–624.

56. T. Q. Dinh, J. I. Yoon, J. Marco, P. Jennings, K. K. Ahn, C. Ha, Sensorless force feedback joystick control for teleoperation of construction equipment. *Int. J. Precis. Eng. Manuf.* **18**, 955–969 (2017).

57. S. Li, R. Rameshwar, A. M. Votta, C. D. Onal, Intuitive control of a robotic arm and hand system with pneumatic haptic feedback. *IEEE Robot. Autom. Lett.* **4**, 4424–4430 (2019).

58. D. Leonardis, M. Gabardi, S. Marcheschi, M. Barsotti, F. Porcini, D. Chiaradia, A. Frisoli, Hand teleoperation with combined kinaesthetic and tactile feedback: A full upper limb exoskeleton interface enhanced by tactile linear actuators. *Robotics* **13**, 119 (2024).

59. K. Fujimoto, F. Kobayashi, H. Nakamoto, F. Kojima, "Development of haptic device for five-fingered robot hand teleoperation" in *Proceedings of the 2013 IEEE/SICE International Symposium on System Integration* (IEEE, 2013; https://ieeexplore.ieee.org/document/6776746), pp. 820–825.

60. D. S. Pamungkas, K. Ward, "Tele-operation of a robot arm with electro tactile feedback" in *2013 IEEE/ASME International Conference on Advanced Intelligent Mechatronics* (IEEE, 2013; https://ieeexplore.ieee.org/document/6584175), pp. 704–709.
